# VNRX-5133 (Taniborbactam), a Broad-Spectrum Inhibitor of Serine- and Metallo-β-Lactamases, Restores Activity of Cefepime in *Enterobacterales* and Pseudomonas aeruginosa

**DOI:** 10.1128/AAC.01963-19

**Published:** 2020-02-21

**Authors:** Jodie C. Hamrick, Jean-Denis Docquier, Tsuyoshi Uehara, Cullen L. Myers, David A. Six, Cassandra L. Chatwin, Kaitlyn J. John, Salvador F. Vernacchio, Susan M. Cusick, Robert E. L. Trout, Cecilia Pozzi, Filomena De Luca, Manuela Benvenuti, Stefano Mangani, Bin Liu, Randy W. Jackson, Greg Moeck, Luigi Xerri, Christopher J. Burns, Daniel C. Pevear, Denis M. Daigle

**Affiliations:** a Venatorx Pharmaceuticals Incorporated, Malvern, Pennsylvania, USA; b Department of Medical Biotechnology, University of Siena, Siena, Italy; c Department of Biotechnology, Chemistry and Pharmacy, University of Siena, Siena, Italy

**Keywords:** antibacterial, β-lactamases, β-lactams, biochemistry, microbiology, structural biology

## Abstract

As shifts in the epidemiology of β-lactamase-mediated resistance continue, carbapenem-resistant *Enterobacterales* (CRE) and carbapenem-resistant Pseudomonas aeruginosa (CRPA) are the most urgent threats. Although approved β-lactam (BL)–β-lactamase inhibitor (BLI) combinations address widespread serine β-lactamases (SBLs), such as CTX-M-15, none provide broad coverage against either clinically important serine-β-lactamases (KPC, OXA-48) or clinically important metallo-β-lactamases (MBLs; e.g., NDM-1). VNRX-5133 (taniborbactam) is a new cyclic boronate BLI that is in clinical development combined with cefepime for the treatment of infections caused by β-lactamase-producing CRE and CRPA. Taniborbactam is the first BLI with direct inhibitory activity against Ambler class A, B, C, and D enzymes. From biochemical and structural analyses, taniborbactam exploits substrate mimicry while employing distinct mechanisms to inhibit both SBLs and MBLs. It is a reversible covalent inhibitor of SBLs with slow dissociation and a prolonged active-site residence time (half-life, 30 to 105 min), while in MBLs, it behaves as a competitive inhibitor, with inhibitor constant (*K_i_*) values ranging from 0.019 to 0.081 μM. Inhibition is achieved by mimicking the transition state structure and exploiting interactions with highly conserved active-site residues. In microbiological testing, taniborbactam restored cefepime activity in 33/34 engineered Escherichia coli strains overproducing individual enzymes covering Ambler classes A, B, C, and D, providing up to a 1,024-fold shift in the MIC. Addition of taniborbactam restored the antibacterial activity of cefepime against all 102 *Enterobacterales* clinical isolates tested and 38/41 P. aeruginosa clinical isolates tested with MIC_90_s of 1 and 4 μg/ml, respectively, representing ≥256- and ≥32-fold improvements, respectively, in antibacterial activity over that of cefepime alone. The data demonstrate the potent, broad-spectrum rescue of cefepime activity by taniborbactam against clinical isolates of CRE and CRPA.

## Abstract (precis)

As shifts in the epidemiology of β-lactamase-mediated resistance continue, carbapenem-resistant *Enterobacterales* (CRE) and carbapenem-resistant Pseudomonas aeruginosa (CRPA) are the most urgent threats.

## INTRODUCTION

There is an urgent need for new therapies to address the rise of infections caused by multidrug-resistant (MDR) Gram-negative bacteria. Of particular concern are health care-associated infections caused by *Enterobacterales* and Pseudomonas aeruginosa, in which acquired resistance to reserved carbapenems significantly narrows the therapeutic options (1–4). According to a 2019 CDC report, in the United States between 2012 and 2017, there were 210,500 cases per year of infections caused by extended-spectrum-β-lactamase (ESBL)-producing *Enterobacterales* or carbapenem-resistant *Enterobacterales* (CRE) and 6,700 cases per year of infections caused by multidrug-resistant P. aeruginosa, resulting in 12,900 deaths annually (5). A 2017 study estimated the resistance burden within U.S. inpatients to be 290,000 ESBL-producing, 170,000 MDR, and 30,000 carbapenem-nonsusceptible *Enterobacterales* infections per year (6). No new antibiotic classes, particularly those active against the challenging Gram-negative pathogens, have been introduced since the introduction of the fluoroquinolones. At the same time, serious resistance and safety concerns over several commonly used classes, including the fluoroquinolones (7–9) and polymyxins (10–11), have severely narrowed safe and effective options for the treatment of these life-threatening infections (4).

β-Lactams (BLs; e.g., penicillins, cephalosporins, monobactams, and carbapenems) are the standard of care for most Gram-negative bacterial infections (12). However, the rate of resistance conferred by β-lactamases continues to increase. More than 2,800 unique β-lactamases that span the spectrum of Ambler classes (Ambler classes A, B, C, and D) have been identified, therein threatening the efficacy of β-lactams (13–14). Key among these are the carbapenem-inactivating serine β-lactamases (SBLs), including KPC and OXA-48, and the emerging metallo-β-lactamases (MBLs; e.g., NDM and VIM). CRE and carbapenem-resistant Pseudomonas aeruginosa (CRPA) strains producing SBLs and MBLs pose a serious challenge for infectious disease physicians and are a major public health concern (4, 15–18). One effective strategy to address the upsurge of carbapenemases is the use of a combination of a β-lactam (BL) with a β-lactamase inhibitor (BLI) to provide protection from these hydrolyzing enzymes (1, 2, 12). Although recently approved BL-BLI combinations (e.g., ceftazidime-avibactam [19, 20], ceftolozane-tazobactam [20], and meropenem-vaborbactam [21]) do offer protection from many SBLs, there are no approved BL-BLI combinations that are active against emerging metallo-β-lactamases (22–24). As for SBL producers, recent ceftazidime-avibactam and ceftolozane-tazobactam treatment failures of infections caused by Klebsiella pneumoniae or P. aeruginosa resulting from the production of KPC-3 or *Pseudomonas*-derived cephalosporinase (PDC) variants highlight the need for new agents that provide a broader spectrum of coverage (25–28). Early cyclic boronate inhibitors were demonstrated to have a significant potential to inhibit all classes of β-lactamases to enable improved broad-spectrum coverage (29, 30).

**FIG 1:**
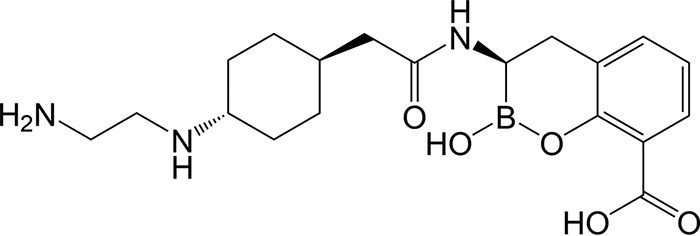
Structure of taniborbactam (VNRX-5133).

A Venatorx Pharmaceuticals patent published in 2014 first disclosed the cyclic boronate BLI taniborbactam (formerly VNRX-5133) (Fig. 1) (31), whereas the discovery and medicinal chemistry optimization of taniborbactam were recently described (32). We present herein comprehensive biochemical, structural, and microbiological data describing the broad-spectrum activity of taniborbactam in combination with the fourth-generation cephalosporin cefepime and compare those data to data for recently approved cephalosporin-BLI combinations. Our findings provide both the biochemical and structural bases for the broad-spectrum inhibition of β-lactamases by taniborbactam and show that addition of this next-generation BLI restores the antibacterial activity of cefepime against *Enterobacterales* and P. aeruginosa producing clinically important SBLs and MBLs, including CTX-M-, KPC-, OXA-, NDM-, and VIM-type β-lactamases.

## RESULTS

### Biochemical and structural studies of taniborbactam, defining the mechanism of inhibition of both SBLs and MBLs.

The kinetic parameters of taniborbactam (Fig. 1) relative to those of avibactam and vaborbactam, including the rate of covalent bond formation (*k*_2_/*K_i_*), off rates (*k*_off_), and the half-lives of active-site occupancy (*t*_1/2_) with CTX-M-15 (class A), KPC-2 (class A), and P99 AmpC (class C) are presented in Table 1. The inhibition behavior of all three BLIs with SBLs fits a two-step inhibition model, in which a noncovalent complex forms, followed by the formation of a reversible covalent bond with the active-site serine residue (equation 1).

(1) $$E+I\;\overset{k_{1}}{\underset{k_{-1}}{⇄}}\;\mathrm{EI}\;\overset{k_{2}}{\underset{k_{-2}}{⇄}}\;\mathrm{EI}*$$

where *E* is enzyme, *I* is inhibitor, and *k*_1_ is the rate of association of the noncovalent Michaelis-Menten complex, *k*_2_ is the rate of formation of the covalent bond, *k*_−2_ is the off rate, and EI* is the covalent enzyme-inhibitor complex. The second-order rate constants (*k*_2_*/K_i_*, where *K_i_* is the inhibitor constant) of covalent bond formation to the active-site serine of the three β-lactamases examined (CTX-M-15, P99 AmpC, and KPC-2) were on the order of 10^4^ to 10^5^ M^−1^ s^−1^ for taniborbactam, whereas they were 10^3^ to 10^5^ for avibactam and 10^3^ for vaborbactam (Table 1). Due to slow inhibitor off rates (*k*_off_ value range, 1.1 × 10^−4^ to 3.8 × 10^−4^ s^−1^), taniborbactam exhibited a significant residence time within the active site, with the *t*_1/2_ values ranging from 30 to 105 min, whereas the *t*_1/2_ values ranged from 29 to 249 min for avibactam, consistent with published data (33), and from 5 to 32 min for vaborbactam (Table 1).

**TABLE 1:** Kinetic parameters of reversible inactivation of serine β-lactamases by taniborbactam

β-Lactamase inhibitor	Kinetic parameter	Value for the following β-lactamases:		
		CTX-M-15	KPC-2	P99 AmpC
Taniborbactam	*k*_2_*/K_i_* (10^4^ M^−1^ s^−1^)	2.1 ± 0.1	0.9 ± 0.1	17.2 ± 0.8
	*k*_off_ (10^−4^ s^−1^)	3.4 ± 0.2	1.1 ± 0.1	3.8 ± 0.3
	*t*_1/2_ (min)	34 ± 2	105 ± 5	30 ± 3
Avibactam	*k*_2_*/K_i_* (10^4^ M^−1^ s^−1^)	10.8 ± 0.6	1.2 ± 0.1	0.32 ± 0.01
	*k*_off_ (10^−4^ s^−1^)	4 ± 0.1	1.8 ± 0.1	0.5 ± 0.04
	*t*_1/2_ (min)	29 ± 1	66 ± 4	249 ± 19
Vaborbactam	*k*_2_*/K_i_* (10^4^ M^−1^ s^−1^)	0.11 ± 0.01	0.12 ± 0.01	0.18 ± 0.01
	*k*_off_ (10^−4^ s^−1^)	23 ± 0.9	5.4 ± 0.5	3.7 ± 0.3
	*t*_1/2_ (min)	5 ± 0.2	21 ± 2	32 ± 3

**FIG 2:**
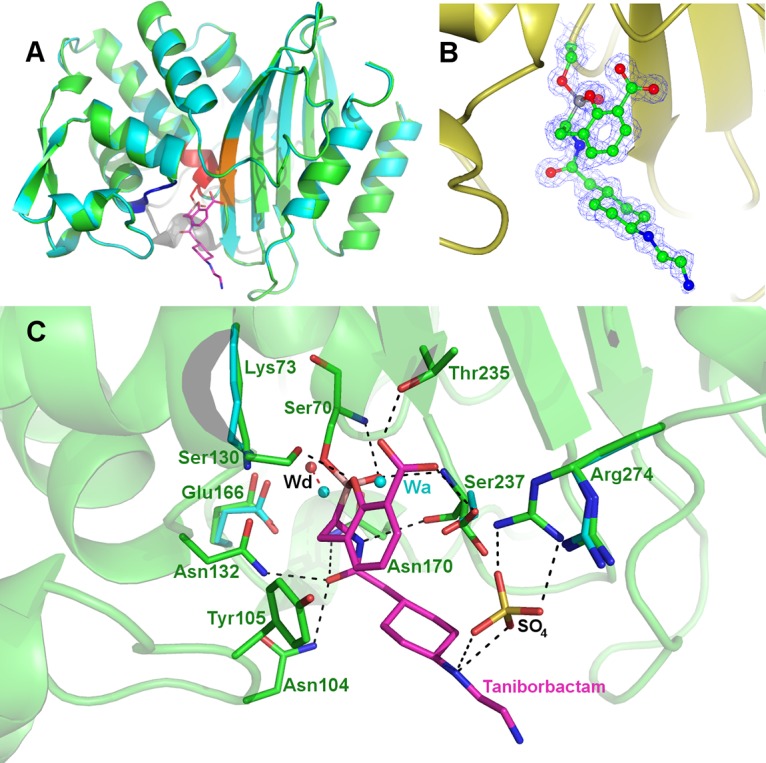
(A) Overall fold of the superimposed native CTX-M-15 (cyan; PDB accession number 4HBT) (55) and its covalent complex with taniborbactam (green; PDB accession number 6SP6) (32). (B) Active-site close-up and omit map of the taniborbactam-bound CTX-M-15 complex. (C) Mode of binding of taniborbactam in the active site of the class A ESBL CTX-M-15, showing the main interactions between the enzyme and taniborbactam (magenta); taniborbactam interacts with many conserved residues of serine-β-lactamases (Asn104, Ser130, Asn132, Asn170, Thr235); compared to the structure of the native CTX-M-15 (PDB accession number 4HBT), the deacylation water molecule (Wd) is displaced by 1.4 Å upon inhibitor binding. Wa refers to acylation water, and SO_4_ is the sulfate from the crystallization buffer solution. Figures were prepared with the CCP4mg (56) or PyMOL (https://pymol.org) program.

A 1.1-Å resolution X-ray cocrystal structure of taniborbactam and CTX-M-15 (PDB accession number 6SP6) (32) previously confirmed that the inhibitor binds covalently to the catalytic Ser70 with a bond distance of 1.53 Å and that the boron atom adopts a tetrahedral conformation (Fig. 2). The location of the boron hydroxyl group in the oxyanion hole suggests that the inhibitor acts as a mimetic of the tetrahedral intermediate formed during the acylation step. Additionally, the binding of taniborbactam displaces the deacylation water molecule (Wd) by 1.4 Å (Fig. 2C) and exploits substrate-like interactions with conserved active-site residues in serine-β-lactamases (Asn104, Ser130, Asn132, Asn170, and Thr235) (32).

**FIG 3:**
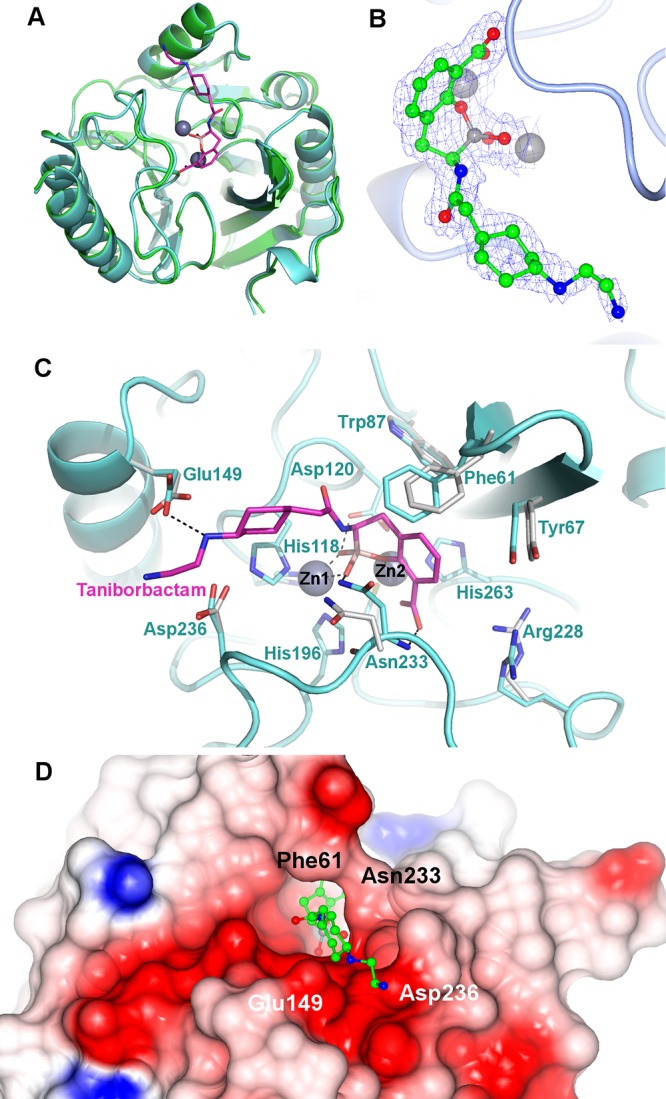
(A) Overall fold of the superimposed native VIM-2 metallo-β-lactamase (cyan; PDB accession number 1KO3) (34) and its covalent complex with taniborbactam (green; PDB accession number 6SP7) (32). (B) Active-site close-up and omit map of the taniborbactam-bound VIM-2 complex. (C) Mode of binding of taniborbactam in the active site of the VIM-2 metallo-β-lactamase, showing the main interactions between the enzyme and VNRX-5133 (magenta). Residues were numbered according to the BBL consensus numbering scheme (57). (D) Surface rendering of the VIM-2 active site in the VNRX-5133-inhibited complex, showing an inhibitor-induced narrowing of the active-site cleft resulting from a closer contact between the side chains Phe61 and Asn233. The presence of the electronegative pocket interacting with the inhibitor side chain and contributing to the stability of the inhibitor-enzyme complex is also shown.

Taniborbactam is distinguished from avibactam and vaborbactam by the ability to inhibit the most clinically relevant subclass B1 MBLs (VIM- and NDM-type enzymes). As observed in the cocrystal structure of taniborbactam and VIM-2 (PDB accession number 6SP7) (Fig. 3) (32), the boron atom adopts an *sp*^3^ hybridization state due to geminal diol formation after reacting with the active-site hydroxide anion (the so-called bridging water coordinated to the Zn^2+^ active-site cations). The boron hydroxyl interacts with Zn-1 and both the conserved Asn233 and Asp120 residues. The carboxylate and oxygen atom of the cyclic oxaborinane interacts with Zn-2, thus behaving as a mimetic of the tetrahedral intermediate in subclass B1 enzymes. The substituted amino group of the inhibitor side chain interacts with Glu149, which is conserved in NDM-1. Interestingly, the inhibitor carboxylate does not interact with Arg228 but instead interacts with the backbone of conserved Asn233. By comparing the bound structure with that for apoVIM-2 (PDB accession number 1KO3) (34), the binding of taniborbactam induces a narrowing of the active-site cleft due to the approach of conserved Asn233 and Phe61 (Fig. 3C). Finally, a surface rendering of VIM-2 bound by taniborbactam shows the presence of an electronegative pocket stabilizing the inhibitor side chain (Fig. 3D), also providing a structural basis for inhibition of both VIM- and NDM-type MBLs, as residues constituting the pocket (Glu149 and Asp236) are conserved in both enzyme subgroups.

The inhibition of VIM-2 and NDM-1 by taniborbactam was reversible, as enzymatic activity was fully recovered after a rapid jump dilution. Using steady-state kinetic analysis, taniborbactam was confirmed to be a competitive inhibitor of VIM-2 and NDM-1 with *K_i_* values of 0.019 and 0.081 μM respectively, whereas inhibition of IMP-1 was outside of the spectrum of inhibition, with *K_i_* being greater than 30 μM (Table 2). Regarding the serine β-lactamases, taniborbactam had potent inhibitory activity against class A and C enzymes, with *K_i_* values ranging from 0.002 to 0.017 μM for SHV-5, KPC-2, CTX-M-15, and P99 AmpC, similar to the values for avibactam. Against the class D OXA-48 enzyme, taniborbactam had a *K_i_* of 0.35 μM, similar to the values for avibactam and vaborbactam (Table 2), and that level of potency was sufficient to protect cefepime from this β-lactamase subtype, which exhibits weak cefepimase activity and, consequently, which contributes minimally to cefepime resistance (Table 3) (35, 36).

**TABLE 2:** *K_i_* values for taniborbactam with various β-lactamases

β-Lactamase	Class	*K_i_* (μM)^*a*^		
		Taniborbactam	Avibactam	Vaborbactam
SHV-5	A	0.003 ± 0.0002	ND	ND
CTX-M-15	A	0.017 ± 0.002	0.011 ± 0.001	0.158 ± 0.006
KPC-2	A	0.004 ± 0.001	0.0056 ± 0.0007	0.022 ± 0.002
				
NDM-1	B	0.081 ± 0.003	>30	>30
VIM-2	B	0.019 ± 0.001	>30	>30
IMP-1	B	>30	>30	>30
				
P99 AmpC	C	0.002 ± 0.0003	0.013 ± 0.0003	0.053 ± 0.004
				
OXA-48	D	0.35 ± 0.007	0.26 ± 0.005	0.35 ± 0.007

a $K_{i}^{*}$ values are used for Ambler class A, C, and B enzymes, and *K_i_* values are used for Ambler class B enzymes, as described in Materials and Methods. ND, not determined.

**TABLE 3:** Spectrum of antibacterial activity of cefepime-taniborbactam defined in engineered strains of E. coli producing individual class A, B, C, and D β-lactamases^*a*^

E. coli DH5α/pTU501 expression	Ambler class	Parameter of antibacterial activity for:					
		CAZ and CZA			FEP and TAN		
		MIC (μg/ml)		Fold potentiation of CAZ activity	MIC (μg/ml)		Fold potentiation of FEP activity
		CAZ	CZA		FEP	FEP-TAN	
Vector control	NA	0.5	0.25	2	0.12	0.12	1
							
TEM-10	A	1,024	2	512	8	0.25	32
TEM-24	A	1,024	16	64	2	0.25	8
TEM-72	A	1,024	1	1,024	32	0.5	64
CTX-M-2	A	32	0.5	64	128	0.25	512
CTX-M-15	A	128	1	128	128	0.25	512
GES-5	A	64	4	16	4	0.25	16
SHV-5	A	1,024	4	256	128	0.12	1,024
VEB-9	A	1,024	16	64	128	0.5	256
KPC-2	A	64	1	64	64	0.12	512
KPC-3	A	512	4	128	128	0.25	512
KPC-3(D179Y)	A	1,024	128	8	32	1	32
KPC-3(V240G)	A	1,024	32	32	256	0.5	512
KPC-3(T243A)	A	256	8	32	64	0.12	512
KPC-3(A177E/D179Y)	A	1,024	512	2	16	0.5	32
KPC-3(D179Y/T243M)	A	1,024	256	4	16	0.5	32
PER-1	A	1,024	32	32	512	0.5	1,024
PER-2	A	1,024	128	8	256	0.5	512
							
NDM-1	B	1,024	1,024	1	256	4	64
NDM-5	B	>1,024	>1,024	1	512	4	128
NDM-7	B	>1,024	>1,024	1	512	4	128
VIM-1	B	1,024	1,024	1	128	2	64
VIM-2	B	128	128	1	16	0.12	128
VIM-4	B	256	256	1	32	0.12	256
IMP-1	B	1,024	1,024	1	64	64	1
SPM-1	B	1,024	1,024	1	128	2	64
GIM-1	B	>128	>128	1	4	0.25	16
							
CMY-2	C	512	8	64	2	0.12	16
ACT-C189 (P99 AmpC)	C	256	2	128	8	0.25	32
ACT-17	C	8	0.5	16	4	0.5	8
							
OXA-48	D	1	0.5	2	2	0.12	16
OXA-162	D	2	1	2	8	0.25	32
OXA-163	D	256	4	64	128	0.25	512
OXA-181	D	1	0.5	2	2	0.25	8
OXA-232	D	1	0.5	2	2	0.25	8

a Abbreviations: FEP, cefepime; TAN, taniborbactam; CAZ, ceftazidime; CZA, ceftazidime-avibactam; NA, not applicable. Taniborbactam and avibactam were tested in combination with cefepime and ceftazidime at a fixed concentration of 4 μg/ml each. Modal MIC values from five independent replicates are reported.

### Potency and spectrum of activity defined in engineered Escherichia coli strains overproducing individual β-lactamases.

The spectrum of antibacterial activity of cefepime-taniborbactam and the breadth of inhibitory activity of this next-generation cyclic boronate BLI were assessed in 34 engineered E. coli strains each overproducing an individual β-lactamase and directly compared to those of the clinically approved agent ceftazidime-avibactam. In this manner, the antibacterial activity of the BL-BLI combinations relative to that of the partnered cephalosporins alone provided a quantitative measure of β-lactam potentiation that directly reflects the levels of inhibition of β-lactamase function achieved by addition of BLI. The BLI concentration was fixed at 4 μg/ml, as described in Materials and Methods.

In strains overproducing Ambler class A β-lactamases, addition of taniborbactam potentiated cefepime activity from 8- to 1,024-fold, which is comparable to the level of potentiation of ceftazidime activity by avibactam (Table 3). In 16/17 class A β-lactamase-overproducing strains, cefepime activity was potentiated by taniborbactam to within 4-fold of the activity of cefepime-taniborbactam against the vector-control strain (cefepime-taniborbactam MIC, 0.12 μg/ml), whereas the potentiation of ceftazidime activity by avibactam was found in only 4/17 strains (ceftazidime MIC for the vector-control strain, 0.25 μg/ml). Cefepime-taniborbactam provided potent coverage against clinically derived serine β-lactamases associated with elevated MICs of ceftazidime-avibactam, including strains producing KPC-3 Ω loop variants (D179Y, V240G, A177E/D179Y, and D179Y/T243M) (25, 26), TEM-24, VEB-9, and PER-1 and -2. This side-by-side comparison demonstrated the differentiation of cefepime-taniborbactam from ceftazidime-avibactam through improved class A ESBL and KPC variant coverage by cefepime-taniborbactam.

In strains producing Ambler class B MBLs, the addition of taniborbactam potentiated the antibacterial activity of cefepime by 16- to 256-fold in all class B β-lactamase-producing strains, with the notable exception of strains producing IMP-1, against which taniborbactam had insufficient inhibitory activity (*K_i_* ≥ 30 μM; Table 2) to potentiate the antibacterial activity of cefepime (Table 3). The addition of avibactam failed to restore ceftazidime activity in these strains, as would be expected from its lack of inhibitory activity against MBLs. In contrast, the activity of cefepime against strains overproducing clinically important NDM variants (NDM-1, -5, and -7) was highly potentiated (64- to 128-fold) by the addition of taniborbactam. This analysis therefore established the spectrum of MBL enzymes inhibited by taniborbactam to be SPM-1, GIM-1, and clinically important variants of NDM (NDM-1, -5, and -7) and VIM (VIM-1, -2, and -4).

Among strains producing selected class C enzymes, cefepime-taniborbactam MICs ranged from 0.12 to 0.5 μg/ml, reflecting an 8- to 32-fold potentiation of cefepime activity to within 4-fold of the MIC for the vector-control strain. In contrast, ceftazidime-avibactam MICs were 0.5, 2, and 8 μg/ml for the ACT-17-, ACT-C189-, and CMY-2-overproducing strains, respectively. Although the MIC of 0.5 μg/ml was within 2-fold of that for the vector-control strain, the last two values were 8- and 32-fold higher than the MIC for the vector-control strain for ACT-C189 (P99 AmpC) and CMY-2, respectively, despite avibactam potentiating ceftazidime activity by 64- to 128-fold in these strains.

In strains overproducing class D β-lactamases (OXA-48, OXA-162, OXA-163, OXA-181, and OXA-232), cefepime activity was potentiated 8- to 512-fold by addition of taniborbactam, resulting in MICs of 0.12 to 0.25 μg/ml, which were within 2-fold of the MIC for the vector-control strain. By comparison, addition of avibactam to ceftazidime had a 2-fold potentiation of ceftazidime activity against four of five strains, resulting in MICs of 0.5 to 1 μg/ml, which is in a range similar to the MICs of cefepime-taniborbactam. In the remaining class D β-lactamase-producing strain, OXA-163 exhibited high ceftazidimase activity (ceftazidime MIC, 256 μg/ml), and although ceftazidime activity was potentiated 64-fold by addition of avibactam to an MIC of 4 μg/ml (16-fold higher than the MIC for the vector-control strain), cefepime activity was potentiated 512-fold by the addition of taniborbactam, providing a significantly improved MIC of 0.25 μg/ml, within 2-fold of the MIC for the vector-control strain. From this side-by-side comparison with class D β-lactamase overproducers, cefepime-taniborbactam and ceftazidime-avibactam appear to be equivalently active, though cefepime-taniborbactam exhibited better potency than ceftazidime-avibactam against OXA-163.

Overall, within this panel of 34 distinct β-lactamase-overproducing strains, the potentiation of the cefepime MIC by taniborbactam ranged from 8- to 1,024-fold in 33/34 strains, with a modal potentiation of 512-fold and MIC_50_/MIC_90_ values of 0.25/4 μg/ml, respectively. Thirty-three of 34 (97%) strains had cefepime-taniborbactam MICs of ≤4 μg/ml. The exception was the IMP-1 overproducer (MIC of cefepime alone, 64 μg/ml, with no potentiation by taniborbactam). In contrast, the potentiation of ceftazidime MICs by avibactam ranged from 2- to 1,024-fold in 25/34 strains, with a modal potentiation of 64-fold and MIC_50_/MIC_90_ values of 8/1,024 μg/ml. Nine of 9 (100%) MBL-producing strains and 8 of 25 (32%) SBL-producing strains fell outside of the ceftazidime-avibactam inhibitory spectrum, with MICs of ≥16 μg/ml, providing a measure of differentiation for cefepime-taniborbactam against this challenge set of SBL-overproducing strains encompassing Ambler class A, C, and D β-lactamases.

### Antibacterial activity of cefepime-taniborbactam in reference isolates.

The antibacterial activity of cefepime, cefepime-taniborbactam, and taniborbactam alone against seven publicly available reference type isolates from the CDC, NCTC, and ATCC was assessed (Table 4). This panel encompassed 3 E. coli, 2 K. pneumoniae, and 2 P. aeruginosa isolates and included four quality control (QC) isolates for broth microdilution (37). Of particular note, taniborbactam demonstrated no clinically relevant antibacterial activity (MIC ≥ 512 μg/ml) against the isolates within this panel. Among the β-lactamase-producing *Enterobacterales* isolates, E. coli NCTC 13353 producing CTX-M-15 and K. pneumoniae BAA-1705 producing KPC-2 are routine QC isolates used for the testing of cefepime-taniborbactam (37). Cefepime antibacterial activity was selectively potentiated by addition of 4 μg/ml taniborbactam against isolates producing β-lactamases, with the exception of P. aeruginosa ATCC 27853 (*Pseudomonas*-derived cephalosporinase 5 [PDC-5]), against which cefepime alone was active (Table 4).

**TABLE 4:** Antibacterial activity of cefepime or taniborbactam alone relative to that of the cefepime-taniborbactam combination in quality control reference type isolates^*a*^

Strain	Enzyme content	MIC (μg/ml)		
		FEP	FEP-TAN^*b*^	TAN
E. coli ATCC 25922	None	0.06	0.06	512
E. coli NCTC 13353	CTX-M-15	64	0.25	512
E. coli CDC-0452	NDM	64	0.25	512
K. pneumoniae ATCC 13883	None	0.06	0.06	512
K. pneumoniae BAA 1705	KPC-2	16	0.25	1,024
P. aeruginosa ATCC 27853	PDC-5	1	1	1,024
P. aeruginosa CDC-0457	VIM	16	4	1,024

a Abbreviations: FEP, cefepime; TAN, taniborbactam.

b Taniborbactam was tested in combination with cefepime at a fixed concentration of 4 μg/ml. Modal MIC values are reported. MICs for cefepime-taniborbactam in routine QC isolates E. coli ATCC 25922, E. coli NCTC 13353, K. pneumoniae BAA 1705, and P. aeruginosa ATCC 27853 are within acceptable QC ranges (58).

### Potentiation of cefepime activity by taniborbactam in clinical isolates of *Enterobacterales* and P. aeruginosa.

The antibacterial activity of cefepime-taniborbactam was compared to that of ceftazidime-avibactam, ceftolozane-tazobactam, and cefepime-tazobactam against a diverse panel of *Enterobacterales* and P. aeruginosa clinical isolates with defined β-lactamase subtypes (see Table S1 in the supplemental material). IMP-producing strains were excluded, as none of the BLI-protected cephalosporins inhibited IMP sufficiently to provide a clinically relevant rescue of the partner β-lactam, a characteristic also shared by approved BLI-protected carbapenems (meropenem-vaborbactam and imipenem-relebactam). The panel does not reflect current epidemiological trends but instead highlights the differences in coverage provided by cefepime-taniborbactam from that provided by the comparators. The antibacterial activity of the cephalosporins alone was included to ascertain the level of potentiation by the partnered BLI. BLIs were tested at a fixed concentration of 4 μg/ml in all combinations except cefepime-tazobactam, where tazobactam was fixed at a concentration of 8 μg/ml (38). The collection comprised 143 clinical isolates collected from 2005 and 2018. Isolates were subdivided by phenotypic profile, with some level of molecular characterization into *Enterobacterales* isolates producing (i) mixed class A and class C β-lactamases and ESBLs (subdivision 1) or (ii) serine- and metallocarbapenemases (OXA-48/48-like, KPC, and NDM/VIM-type MBLs) (subdivision 2) and P. aeruginosa isolates producing (iii) basal levels of PDCs and downregulated OprD combined with upregulated RND drug efflux systems (subdivision 3), (iv) ceftolozane-tazobactam-resistant PDC variants (subdivision 4), or (v) serine and metallocarbapenemases (GES, KPC, VIM) (subdivision 5) (Table S1).

**FIG 4:**
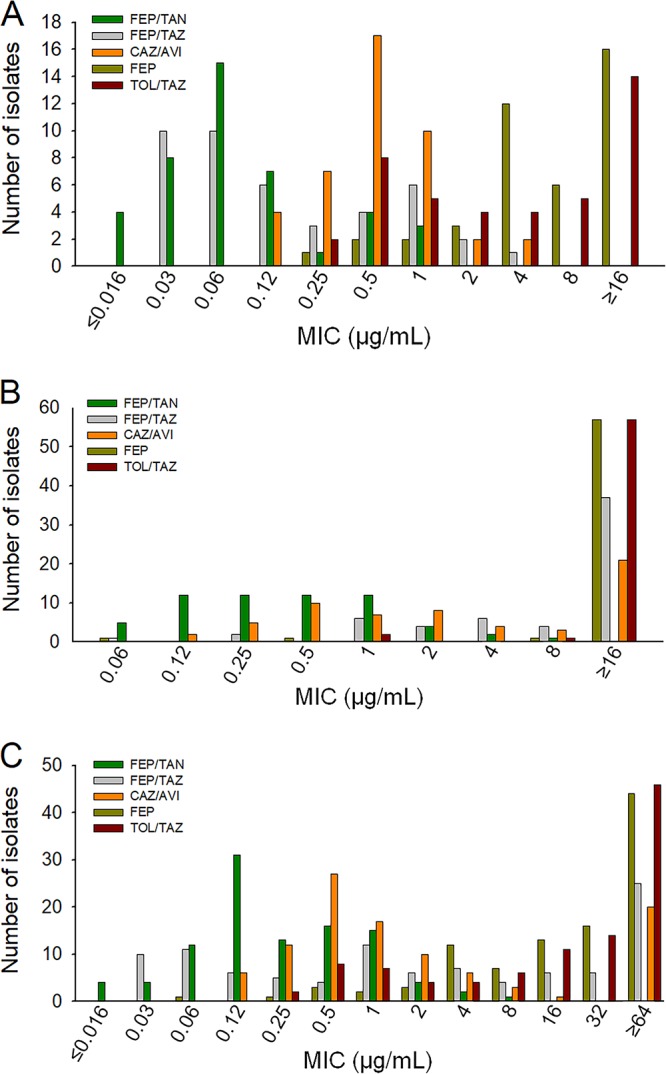
MIC distributions from broth microdilution testing of cefepime-taniborbactam and comparators in *Enterobacterales*. The number of isolates at each MIC for cefepime is shown relative to that for cefepime-tazobactam, cefepime-taniborbactam, ceftazidime-avibactam, and ceftolozane-tazobactam, with the concentration of the BLI being fixed at 4 μg/ml for all combinations except cefepime-tazobactam, for which the concentration of the BLI was fixed at 8 μg/ml. Susceptibility was defined as an MIC of ≤8 μg/ml for cefepime alone, cefepime-tazobactam, cefepime-taniborbactam, and ceftazidime-avibactam or an MIC of ≤2 μg/ml for ceftolozane-tazobactam in *Enterobacterales*. (A) *Enterobacterales* producing mixed class A and C and extended-spectrum β-lactamases (*n* = 42). The MIC_50_/MIC_90_ values were 8/128, 0.12/1, 0.06/0.5, 0.5/1, and 4/32 μg/ml for cefepime, cefepime-tazobactam, cefepime-taniborbactam, ceftazidime-avibactam, and ceftolozane-tazobactam, respectively. Percent susceptibility to these drugs was 61.9%, 100%, 100%, 100%, and 42.9%, respectively. (B) *Enterobacterales* producing carbapenemases, including OXA-48/OXA-48-like and KPC β-lactamases and metallo-β-lactamases (*n* = 60). The MIC_50_/MIC_90_ values were 64/≥256, 32/≥64, 0.5/2, 2/≥64, and ≥64/≥64 μg/ml for cefepime, cefepime-tazobactam, cefepime-taniborbactam, ceftazidime-avibactam, and ceftolozane-tazobactam, respectively. Percent susceptibility to these drugs was 5%, 38.3%, 100%, 65%, and 3.3%, respectively. (C) Overall distribution of MICs in all 102 isolates of *Enterobacterales* class A and C, OXA-48/OXA-48-like β-lactamases, ESBLs, and KPC, VIM-type, and NDM-type β-lactamases. The MIC_50_/MIC_90_ values were 32/≥256, 2/≥64, 0.12/1, 1/≥64, and 32/≥64 μg/ml for cefepime, cefepime-tazobactam, cefepime-taniborbactam, ceftazidime-avibactam, and ceftolozane-tazobactam, respectively. Percent susceptibility to these drugs was 28.4%, 63.7%, 100%, 79.4%, and 19.6%, respectively. FEP, cefepime; FEP/TAZ, cefepime-tazobactam; FEP/TAN, cefepime-taniborbactam; CAZ/AVI, ceftazidime-avibactam; TOL/TAZ, ceftolozane-tazobactam.

In 42 isolates of *Enterobacterales* (subdivision 1) expressing either mixed class A and C β-lactamases or ESBLs, cefepime-taniborbactam, cefepime-tazobactam, and ceftazidime-avibactam had similar levels of activity, with MIC_50_/MIC_90_ values of 0.06/0.5, 0.12/1, and 0.5/1 μg/ml, respectively, whereas the MIC_50_/MIC_90_ values of ceftolozane-tazobactam were 4/32 μg/ml for this set of isolates (Fig. 4A; Table S1).

In 60 *Enterobacterales* isolates (subdivision 2) expressing carbapenemases (OXA-48/48-like, KPC, or NDM-/VIM metallo-β-lactamases), cefepime-taniborbactam was highly active, with an MIC_50_/MIC_90_ of 0.5/2 μg/ml, relative to the activities of the comparators ceftazidime-avibactam (MIC_50_/MIC_90_, 2/≥64 μg/ml), cefepime-tazobactam (MIC_50_/MIC_90_, 32/≥64 μg/ml), cefepime alone (MIC_50_/MIC_90_, 64/≥64 μg/ml), and ceftolozane-tazobactam (MIC_50_/MIC_90_, ≥64/≥64 μg/ml) (Fig. 4B; Table S1).

The overall activity of the tested agents against 102 *Enterobacterales* isolates is summarized in Fig. 4C and Table S1. The distributions of the MICs of cefepime alone along with those of the four cephalosporin-BLI combinations are presented. Addition of taniborbactam reduced the MIC_90_ from ≥256 μg/ml for cefepime alone to 1 μg/ml (Table S1). Cefepime-taniborbactam was the most active combination, followed by ceftazidime-avibactam, with MIC_50_/MIC_90_ values of 0.12/1 and 1/≥64 μg/ml, respectively (Fig. 4C; Table S1).

**FIG 5:**
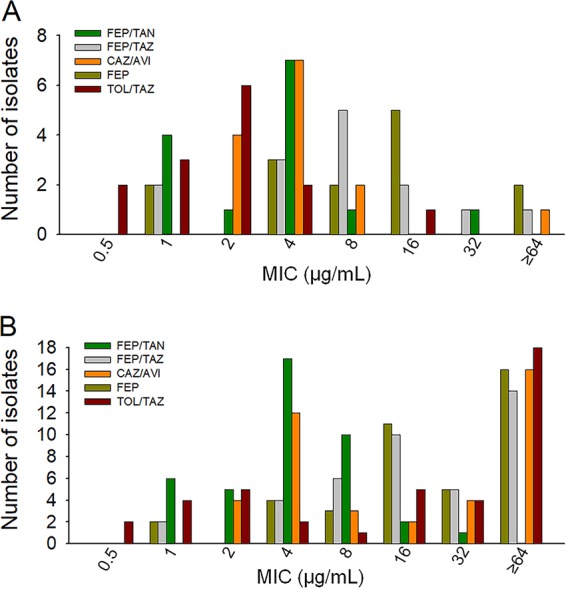
MIC distributions from broth microdilution testing in P. aeruginosa. The number of isolates at each MIC for cefepime is shown relative to that for cefepime-tazobactam, cefepime-taniborbactam, ceftazidime-avibactam, and ceftolozane-tazobactam, with the concentration of the BLI being fixed at 4 μg/ml for all combinations except cefepime-tazobactam, for which the concentration of the BLI was fixed at 8 μg/ml. Susceptibility was defined as an MIC of ≤8 μg/ml for cefepime alone, cefepime-tazobactam, cefepime-taniborbactam, and ceftazidime-avibactam or an MIC ≤4 μg/ml for ceftolozane-tazobactam in P. aeruginosa. (A) P. aeruginosa with wild-type basal PDC expression or downregulated OprD combined with upregulated RND drug efflux systems and PDC variant expression levels (*n* = 14). MIC_50_/MIC_90_ values were 8/≥64, 8/32, 4/8, 4/8, and 2/4 μg/ml for cefepime, cefepime-tazobactam, cefepime-taniborbactam, ceftazidime-avibactam, and ceftolozane-tazobactam, respectively. Percent susceptibility to these drugs was 50%, 71.4%, 92.9%, 92.9%, and 92.9%, respectively. (B) Overall distribution of MICs in 41 isolates of P. aeruginosa producing wild-type PDCs with various levels of production of OprD and MexAB-OprM/MexXY-OprM efflux pumps, PDC variants affecting the activity of ceftolozane-tazobactam, and KPC, GES, or VIM carbapenemases. The MIC_50_/MIC_90_ values were 32/≥256, 16/≥64, 4/8, 16/≥64, and 32/≥64 μg/ml for cefepime, cefepime-tazobactam, cefepime-taniborbactam, ceftazidime-avibactam, and ceftolozane-tazobactam, respectively. Percent susceptibility to these drugs was 22%, 29.3%, 92.7%, 46.3%, and 31.7%, respectively. FEP, cefepime; FEP/TAZ, cefepime-tazobactam; FEP/TAN, cefepime-taniborbactam; CAZ/AVI, ceftazidime-avibactam; TOL/TAZ, ceftolozane-tazobactam.

Against 14 Pseudomonas aeruginosa isolates (subdivision 3), ceftolozane-tazobactam (MIC_90_ = 4 μg/ml), cefepime-taniborbactam (MIC_90_ = 8 μg/ml), and ceftazidime-avibactam (MIC_90_ = 8 μg/ml) were all highly active. Cefepime-tazobactam was less active than the other combinations against these isolates, with an MIC_90_ of 32 μg/ml (Fig. 5A). The addition of taniborbactam reduced the cefepime MIC_90_ by ≥8-fold for this subset of isolates, similar to the potentiation of ceftazidime activity by avibactam. In contrast, the addition of tazobactam reduced the MIC_90_ of ceftolozane by 4-fold and that of cefepime by 2-fold. Of note, one isolate in this subset, Paeβ-18, upregulated both MexAB-OprM (3.9-fold) and MexXY (5.6-fold), combined with a 600-fold upregulation of PDC-3 expression, resulting in MICs of 16, 32, and ≥64 μg/ml for ceftolozane-tazobactam, cefepime-taniborbactam, and ceftazidime-avibactam, respectively (39). The data suggest that the combined effect of drug efflux upregulation and highly elevated PDC variant production in P. aeruginosa can effectively reduce susceptibility to these cephalosporin-BLI combinations (Table S1).

Among 10 isolates of P. aeruginosa (subdivision 4) resistant to ceftolozane-tazobactam (MIC, >8 μg/ml) through the production of eight different PDC variants (Table S1), a single isolate remained susceptible to ceftazidime-avibactam with an MIC of 8 μg/ml, while all others had MIC values ranging from 16 to ≥64 μg/ml. Cefepime was more stable against these PDC variants than ceftazidime was, and the combination of cefepime-taniborbactam provided good antibacterial activity with MICs ranging from 2 to 16 μg/ml (Table S1).

Among 17 isolates of P. aeruginosa (subdivision 5) producing KPC (n = 3), GES (n = 9), or VIM (n = 5) carbapenemases, cefepime-taniborbactam was more active than the comparator cephalosporin-BLI combinations. The MIC values of cefepime-taniborbactam for all 17 isolates ranged from 1 to 8 μg/ml, with an MIC_50_ of 4 μg/ml and an MIC_90_ of 8 μg/ml (Table S1). Ceftolozane-tazobactam activity was weak, with MIC values ranging from 16 to ≥64 μg/ml. Ceftazidime-avibactam was active with an MIC of ≤8 μg/ml for 5/17 isolates, including all 3 KPC producers and 2 GES-6 producers, whereas all other isolates exhibited MICs ranging from 16 to ≥64 μg/ml. The data highlight a gap in coverage of the ceftazidime-avibactam combination against GES-producing P. aeruginosa isolates, with 6/9 isolates having MICs of ≥16 μg/ml (Table S1).

Overall, among the 41 P. aeruginosa isolates tested (subdivisions 3 to 5), addition of taniborbactam to cefepime dramatically shifted the distribution of MICs to a lower range, with an MIC_90_ of 8 μg/ml for cefepime-taniborbactam compared to an MIC_90_ of ≥256 μg/ml for cefepime alone (Fig. 5B; Table S1). Ceftazidime-avibactam and ceftolozane-tazobactam had elevated MIC_90_ values of ≥64 μg/ml due to the production of VIM and GES variants along with cross-resistance to ceftazidime-avibactam among the ceftolozane-tazobactam-resistant isolates, consistent with previously published findings (27, 28).

### Addition of taniborbactam restores the bactericidal activity of cefepime in NDM-1-producing K. pneumoniae and VIM-2-producing P. aeruginosa clinical isolates.

**FIG 6:**
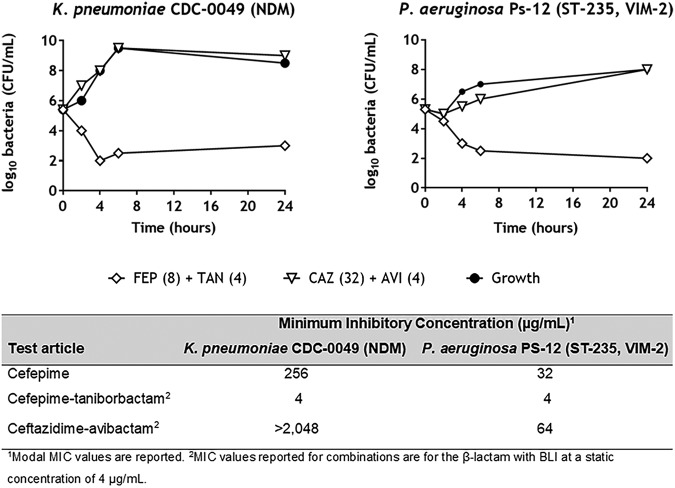
Time-kill curves for cefepime-taniborbactam relative to those for ceftazidime-avibactam in two metallo-β-lactamase-producing clinical isolates. The log_10_ value of the number of viable CFU per milliliter is displayed on the *y* axis versus time (in hours) on the *x* axis. Curves for 1×, 2×, and 4× MIC for cefepime (FEP) with taniborbactam (TAN) fixed at 4 μg/ml are shown for each strain, while the curves for ceftazidime (CAZ) at 32 μg/ml with avibactam (AVI) fixed at 4 μg/ml, representing 0.015× MIC (K. pneumoniae CDC-0049) and 0.5× MIC (P. aeruginosa PS-12) of ceftazidime, which are well above the CLSI susceptibility breakpoint for ceftazidime-avibactam in *Enterobacterales* and P. aeruginosa of 8 μg/ml, are shown for each strain.

Restoration of the bactericidal activity to cefepime by addition of taniborbactam was further confirmed by time-kill assays with K. pneumoniae CDC-0049 producing NDM-1 and P. aeruginosa Ps-12 producing VIM-2 (Fig. 6). Both isolates were resistant to cefepime, with MICs of 256 μg/ml and 32 μg/ml, respectively. Addition of taniborbactam at a fixed concentration of 4 μg/ml shifted the cefepime MIC to 4 μg/ml in both cases. In both isolates, cefepime-taniborbactam achieved a 3-log_10_ reduction in the number of CFU per milliliter relative to the starting inoculum by 6 h, without regrowth thereafter. In contrast, ceftazidime-avibactam tested at 32 μg/ml (4-fold above the CLSI susceptibility breakpoint of 8 μg/ml) had little to no impact on either isolate due to the high MICs originating from a lack of coverage of VIM- and NDM-type MBLs.

### Resistance to cefepime-taniborbactam.

Frequency-of-resistance (FoR) studies were performed at 4× MIC of cefepime with taniborbactam fixed at 4 μg/ml against 8 *Enterobacterales* and P. aeruginosa strains expressing Ambler class A, B, C, and D β-lactamases. The FoR ranged from 1.6 × 10^−9^ to 8.9 × 10^−11^ (Table 5), indicating a low potential for the spontaneous development of resistance to cefepime-taniborbactam among the target pathogens. Moreover, no single-step taniborbactam-resistant β-lactamase variants were obtained from this standard first-pass investigation.

**TABLE 5:** Spontaneous frequency of resistance to cefepime-taniborbactam in select Gram-negative bacteria

Strain	Enzyme content(s)	MIC^*a*^ (μg/ml)	Total no. of CFU	FoR^*b*^
E. coli 25922	AmpC	0.03	2.19 × 10^10^	9.1 × 10^−11^
P. aeruginosa 27853	PDC-5	2	2.5 × 10^9^	<4 × 10^−10^
K. pneumoniae BAA 1705	KPC-2	0.125	4.6 × 10^9^	6.5 × 10^−10^
E. cloacae ECL01	P99 AmpC	0.06	1.31 × 10^10^	8.9 × 10^−11^
E. coli ESBL 4	CTX-M-15, TEM-1	0.06	4.1 × 10^9^	2.4 × 10^−10^
K. pneumoniae SI-117	VIM-1	0.25	2.4 × 10^9^	<4.2 × 10^−10^
E. coli SI-152	NDM-1	0.125	9 × 10^9^	1.6 × 10^−9^
E. coli VER	OXA-48	0.03	4.8 × 10^9^	4.2 × 10^−10^

a The MIC of cefepime titrated with taniborbactam fixed at 4 μg/ml.

b Agar plates contained cefepime at 4× MIC and taniborbactam fixed at 4 μg/ml. FoR was calculated as (number of CFU observed in the presence of cefepime-taniborbactam)/(total number of CFU).

### High selectivity for β-lactamases.

The selectivity and specificity of taniborbactam were evaluated in the DrugMatrixScreen panel of pharmacological targets at Eurofins/Panlabs. Binding, enzymatic, and uptake assays representing a wide range of cellular and subcellular target classes were performed. At the screening concentration of 100 μM, no notable off-target findings (≤23% inhibition) were reported in 128 of 129 *in vitro* assays. The one exception in which significant inhibition (>50%) was observed was with a β-lactamase.

## DISCUSSION

The goal of a β-lactamase inhibitor in a BL-BLI combination is to rescue the β-lactam from degradation by the complement of β-lactamases present, thereby restoring its activity to that seen in the absence of β-lactamase enzymes and essentially restoring the MICs to a wild-type distribution. The BL-BLI approach was effectively introduced with the first-generation BLIs tazobactam, sulbactam, and clavulanic acid paired with piperacillin, ampicillin, and amoxicillin, respectively. This strategy has been extended more recently to protected cephalosporins (ceftazidime-avibactam and ceftolozane-tazobactam) and carbapenems (meropenem-vaborbactam and imipenem-relebactam). The focus of these recently approved BL-BLI combinations had been to address the growing concerns regarding KPC-type enzymes. With the advent of protected cephalosporins and carbapenems, there is an opportunity to reestablish the stratification of β-lactams into front-line protected cephalosporins (ceftazidime-avibactam, ceftolozane-tazobactam) and to reserve carbapenems (e.g., meropenem-vaborbactam, imipenem-relebactam) (40). Over the past decade, other problematic β-lactamase expansions in the *Enterobacterales* and P. aeruginosa have emerged, particularly in select geographies, including, most notably, widespread OXA-48/OXA-48-like β-lactamase-producing *Enterobacterales* in Europe (18, 41), VIM-2 MBL-producing sequence type 235 (ST-235) P. aeruginosa strains in Belarus, Kazakhstan, and Russia (42), and NDM-1, -5, and -7 MBL-producing *Enterobacterales* in India and China (43–45). None of the recently approved BL-BLI combinations is sufficiently active against *Enterobacterales* or P. aeruginosa isolates producing MBLs, and meropenem-vaborbactam also lacks coverage against OXA-48 producers (46). Taniborbactam, which is being developed in combination with cefepime for the treatment of complicated urinary tract infections (cUTI) and hospital-acquired or ventilator-associated bacterial pneumonia (HABP/VABP), represents the next stage of this approach. We have now demonstrated the uniquely potent activity of cefepime-taniborbactam against both SBLs and MBLs and especially against these emerging MBLs and OXA-48/OXA-48-like SBLs compared to the activities of ceftazidime-avibactam, ceftolozane-tazobactam, and cefepime-tazobactam.

In addition to emerging MBLs and OXA-48/OXA-48-like SBLs, reports of antibiotic resistance evolving during the course of clinical therapy with BL-BLIs, including resistance to ceftazidime-avibactam in KPC-producing *Enterobacterales* and to ceftolozane-tazobactam in P. aeruginosa strains producing altered PDC variants, are increasing. Most concerning with the PDC variants is the fact that cross-resistance to ceftazidime-avibactam is often associated with resistance to ceftolozane-tazobactam (27, 28). The activity of cefepime-taniborbactam against these resistant variants of PDC and KPC would, importantly, provide a future alternative therapeutic option to address evolving resistance. Cefepime-taniborbactam thus has the potential, once it is approved for clinical use, to provide the broadest coverage of β-lactamases to complement the existing repertoire of agents active against Gram-negative bacteria in the clinic and help address these problematic β-lactamase expansions by providing additional options for physicians to effectively treat these infections.

## MATERIALS AND METHODS

### Expression plasmid construction.

Plasmid DNA, PCR product purification, and gel extractions were performed using Wizard Plus SV miniprep and SV gel and PCR extraction kits (Promega). The NdeI, BamHI, and XhoI restriction enzymes, T4 DNA ligase, and E. coli BL21(DE3) competent cells were purchased from New England Biolabs. All oligonucleotide primers for PCR amplification were purchased from Integrated DNA Technologies. All PCRs were performed with Phusion high-fidelity DNA polymerase, and cloning was performed in subcloning-efficient E. coli DH5α chemically competent cells (Thermo Fisher). The pET9a (Agilent) and pET24a (MilliporeSigma) expression clones were made using PCR amplification products from molecularly characterized clinical isolates carrying the desired β-lactamase gene inserted into pET9a using NdeI and BamHI in all cases except for the *bla*_NDM-1_ gene, for which NdeI and XhoI were used in pET24a. All β-lactamases except NDM-1 were cloned with signal peptide-encoding sequences. NDM-1 lacked the coding sequence for the first 28 amino acids, encompassing the signal peptide and the lipobox (LSGC) peptide sequence. All transformants were verified by PCR amplification, restriction endonuclease mapping, and DNA sequencing. Confirmed expression plasmids were isolated by use of a plasmid miniprep kit and used to transform the expression cell line E. coli BL21(DE3) or E. coli JM109(DE3).

### Construction of isogenic strains of E. coli producing individual β-lactamases.

The isogenic strains were used to establish a spectrum of inhibitory activity against 34 β-lactamases that were engineered into E. coli DH5α cells carrying chromosomal AmpC (ESC-1), a non-ESBL enzyme unable to hydrolyze these third- or fourth-generation cephalosporins. Each β-lactamase gene region encoding the periplasmic protein was placed under the control of the *bla*_TEM-1_ promoter and a signal peptide sequence to drive the expression and localization of the β-lactamases to the periplasm. The DNA fragments containing the promoter, the signal sequence, and each β-lactamase-coding gene were synthesized and cloned into pTwist Chlor High Copy in DH5α at Twist Bioscience. The individual expression plasmids along with their GenBank accession numbers are listed in Table S2 in the supplemental material and below. The protein sequences of β-lactamases were obtained from the Beta-Lactamase DataBase (www.bldb.eu) (47). Expression of the β-lactamases was confirmed by verifying decreased susceptibility to example test antibiotics that are known substrates of the enzymes. E. coli DH5α carrying pTU501, which expressed only the TEM-1 signal sequence from the *bla* promoter, was used as a control. The plasmid sequences are available through GenBank (Table S2).

### β-Lactamase purification.

For all β-lactamases except P99 AmpC, a 50-ml preculture of E. coli BL21(DE3) cells containing the pET-based expression vector (pET24a for NDM-1 and pET9a otherwise) for the individual β-lactamases was grown overnight in lysogeny broth (LB) medium at 37°C in the presence of kanamycin selection at 50 μg/ml. Common to all β-lactamase purifications, bacterial cells were lysed by three consecutive passes through a chilled French pressure cell at 18,500 lb/in^2^ and clarified by centrifugation at 10,000 × *g* for 30 min at 4°C. The β-lactamase activity was monitored using nitrocefin at 100 μM, and purity was examined by 10% SDS-PAGE with Coomassie brilliant blue staining. Purified proteins exhibiting >95% purity by SDS-PAGE were quantified by use of a Pierce bicinchoninic acid protein assay kit and bovine serum albumin (BSA) as the standard (Thermo Fisher), concentrated to a working range of 1 to 5 mg/ml, and frozen at −80°C in buffer containing 10% glycerol. Column purifications were performed using an Åkta fast-performance liquid chromatograph (GE Healthcare). The purification schemes were generally similar, with the enzyme-specific differences being described below.

CTX-M-15 was obtained from E. coli BL21(DE3) carrying plasmid pET-CTX-M-15, grown in 2 liters of MagicMedia autoinduction medium (Invitrogen) containing 50 μg/ml kanamycin (Sigma) for 24 h at 23°C. Cells were harvested at an *A*_600_ of 2.2 by centrifugation at 7,500 × *g* at 4°C and resuspended in 60 ml of 10 mM HEPES, pH 7, supplemented with 0.5 mM EDTA. The lysate was diluted 5-fold with cold 50 mM sodium acetate, pH 4.8, and incubated overnight at 4°C. The extract was clarified by centrifugation at 14,500 × *g* at 4°C, filtered through an Amicon nitrogen concentrator with a 10-kDa-cutoff filter to a volume of 50 ml, and loaded onto a HiTrap Capto S column that had been preequilibrated in 50 mM sodium acetate, pH 4.8. Protein was eluted with a linear gradient of 50 mM sodium acetate, pH 4.8, supplemented with 500 mM NaCl. Fractions containing active CTX-M-15 were pooled, concentrated, and buffer exchanged in 20 mM HEPES, pH 7.2, 150 mM NaCl, and 10% glycerol using Amicon Ultra-15 centrifugal concentrators. CTX-M-15 was further separated by use of a Superdex 200 gel filtration column.

OXA-48 was obtained from E. coli BL21(DE3) carrying plasmid pET-OXA-48, grown in 2 liters of MagicMedia autoinduction medium (Invitrogen) with kanamycin as described above for 24 h at 23°C. Cells were harvested at an *A*_600_ of 3 by centrifugation at 7,500 × *g* at 4°C, resuspended in 60 ml of 20 mM triethanolamine, pH 5.5, and then purified as described above for CTX-M-15, with the exception that the final buffer contained 10 mM NaHCO_3_ to maintain the critical active-site lysine residue carbamylated (48).

KPC-2 was obtained from E. coli BL21(DE3) carrying plasmid pET-KPC-2, grown in 3 liters of MagicMedia autoinduction medium (Invitrogen) with kanamycin as described above for 24 h at 23°C. Cells were harvested at an *A*_600_ of 3.3 by centrifugation at 7,500 × *g* at 4°C and resuspended in 70 ml of 20 mM MES (morpholineethanesulfonic acid; pH 5.5). The extract was clarified by centrifugation at 14,500 × *g* at 4°C, filtered through an Amicon nitrogen concentrator by use of 10-kDa-cutoff filters to a volume of 50 ml, and loaded onto a HiTrap Capto S column that had been preequilibrated in 20 mM MES, pH 5.5. Protein was eluted with a linear gradient of 20 mM MES, pH 5.5, supplemented with 500 mM NaCl. KPC-2 active fractions were pooled, concentrated, and buffer exchanged in 20 mM HEPES, pH 7.3, 150 mM NaCl using Amicon Ultra-15 centrifugal concentrators. KPC-2 was further separated by gel filtration chromatography with a Superdex 200 column.

P99 AmpC was purified directly from the Enterobacter cloacae SIP9925 P99+ clinical isolate after the sequence of the β-lactamase-encoding gene had been verified by DNA sequencing of the PCR-amplified product. To produce P99 AmpC, Enterobacter cloacae P99+ cells were grown in LB in the presence of a sub-MIC (0.015 μg/ml) of imipenem to induce maximal expression of the enzyme. Cells were harvested at an *A*_600_ of 2.4 by centrifugation at 7,500 × *g* at 4°C, resuspended in 50 ml of 20 mM MES, pH 5.0, and otherwise purified in a manner similar to that for KPC-2 described above.

SHV-5 was obtained from E. coli BL21(DE3)/pLysS carrying plasmid pET-SHV-5, grown in 2 liters of Super broth at 25°C to an *A*_600_ of 0.5, when IPTG (isopropyl-β-d-thiogalactopyranoside) was added to a final concentration of 0.05 mM, and induction proceeded for 6 h. Cells were harvested by centrifugation at 5,500 × *g* for 15 min at 4°C. The cell pellet was resuspended in 60 ml of 10 mM HEPES buffer, pH 7.5, and the cells were lysed with a French press. The lysate was diluted 5-fold in 50 mM sodium acetate buffer, pH 5, and kept at 4°C overnight. The extract was clarified by centrifugation at 14,500 × *g* at 4°C, filtered through an Amicon nitrogen concentrator by the use of 10-kDa-cutoff filters to a volume of 50 ml, and loaded onto a HiTrap Capto S column equilibrated with 50 mM sodium acetate buffer, pH 5. SHV-5 was eluted with a linear gradient of 50 mM sodium acetate, pH 5, supplemented with 500 mM NaCl. Active fractions were pooled, concentrated, and buffer exchanged in 20 mM HEPES, pH 7.3, 150 mM NaCl, and 10% glycerol using Amicon Ultra-15 centrifugal concentrators. Finally, the SHV-5 sample was further purified by chromatography over a Superdex 200 gel filtration column.

VIM-2 was purified from E. coli BL21(DE3) carrying pET-VIM-2 as previously described (49) with the following changes. MagicMedia autoinduction medium was used instead of Luria-Bertani (LB) broth with IPTG induction, and the cells were harvested at an *A*_600_ of 2.1 by centrifugation at 7,500 × *g* for 15 min at 4°C. The 50 to 80% ammonium sulfate precipitate was collected by centrifugation at 13,000 × *g* for 1 h at 4°C and solubilized in 20 mM HEPES, pH 7.2, 50 μM ZnSO_4_ at 1/20 of the original volume, and the solution was loaded onto a 30-ml Q Sepharose anion exchange column (GE Healthcare) that had been equilibrated with the same buffer. Elution of VIM-2 was achieved with a linear NaCl gradient to 1 M. Fractions containing VIM-2 were pooled and concentrated with Amicon Ultra-15 centrifugal concentrators with a 10-kDa cutoff. VIM-2 was dialyzed in 50 mM Tris, pH 9, 50 μM ZnSO_4_ overnight and loaded onto a MonoQ column that had been preequilibrated in the same buffer, and elution was achieved with a linear gradient of NaCl up to 500 mM. Active fractions containing VIM-2 were pooled, concentrated, buffer exchanged in 50 mM HEPES, pH 7.5, 50 μM ZnSO_4_, 200 mM NaCl, and further separated by gel filtration with a Superdex 200 column (GE Healthcare) that had been equilibrated in the same buffer.

IMP-1 was purified from E. coli BL21(DE3) carrying plasmid pET-IMP-1, grown in 3 liters of LB at 37°C with 50 μg/ml kanamycin selection as previously described with minor modifications (50). At an *A*_600_ of 0.7, IPTG was added to a final concentration of 0.5 mM, and induction of IMP-1 expression was allowed to proceed for an additional 5 h at 30°C. Cells were centrifuged at 7,500 × *g* for 15 min at 4°C. The pellet was resuspended in 50 mM HEPES, pH 7, containing 50 μM ZnSO_4_. The lysate was clarified by centrifugation at 12,500 × *g* for 30 min at 4°C and loaded onto a HiTrap Capto S column equilibrated in 50 mM HEPES, pH 7, 50 μM ZnSO_4_. The column was washed with the same buffer, and the enzyme was eluted with a linear gradient of NaCl to 500 mM. The active fractions were pooled and concentrated by ultrafiltration with Amicon centrifugal concentrators with a 10-kDa cutoff. The protein solution was dialyzed in 50 mM HEPES, pH 7, 50 μM ZnSO_4_ and loaded onto a MonoS cation exchange column that had been preequilibrated in the same buffer. The enzyme was eluted with a linear gradient of NaCl to 500 mM. Fractions containing active IMP-1 were collected, pooled, and concentrated to 1 mg/ml.

NDM-1 lacking the signal peptide (first 28 amino acids) and lipobox sequence was purified as a soluble protein from the cytoplasm of Escherichia coli JM109(DE3) carrying plasmid pET-NDM-SP, grown in 3 liters of MagicMedia medium with kanamycin selection as described above. Protein expression was induced by the addition of 0.5 mM IPTG at an *A*_600_ of 0.5, followed by incubation at 23°C for 18 h. Cells were harvested by centrifugation at 7,500 × *g* for 15 min at 4°C and washed twice with 25 mM Tris-HCl, pH 7, 50 μM ZnSO_4_. Cell lysates obtained with a French press were clarified by centrifugation at 12,500 × *g* for 30 min at 4°C. The supernatant was loaded onto a Q Sepharose column that had been equilibrated in 25 mM Tris-HCl, pH 7, 50 μM ZnSO_4_, and the enzyme was eluted with a linear gradient of the same buffer supplemented with 500 mM NaCl. Active fractions of NDM-1 were pooled, concentrated, and loaded onto a Superdex 200 gel filtration column that had been equilibrated in 25 mM Tris-HCl, pH 7, 50 μM ZnSO_4_, 150 mM NaCl. NDM-1-containing fractions of high purity were pooled and dialyzed into 10 mM HEPES, pH 7, containing 20 μM ZnSO_4_ and 0.01 mg/ml bovine serum albumin (BSA).

### Reversible inactivation of serine active-site β-lactamases.

The kinetic parameters associated with the reversible inactivation of CTX-M-15, KPC-2, and P99 AmpC were assessed by monitoring CTX-M-15-mediated cephalothin hydrolysis, KPC-2-mediated imipenem hydrolysis, and P99 AmpC-mediated cephalothin hydrolysis spectrophotometrically at 37°C in 50 mM sodium phosphate buffer (pH 7.0) within the first 15 min of the reaction. To measure the hydrolysis rates, we used the following extinction coefficients (Δε): for cephalothin, −6,300 M^−1^ cm^−1^ at 273 nm, and for imipenem, −9,000 M^−1^ cm^−1^ at 299 nm. For KPC-2, reactions with 500-μl reaction mixtures were initiated by addition of 6.25 pmol KPC-2 and were performed in quadruplicate with 75 μM imipenem and six concentrations of taniborbactam (2.5, 3.3, 4, 4.8, 5.5, and 6.3 μM). For P99 AmpC, the reactions were initiated by addition of 1.25 pmol of enzyme and were performed in quadruplicate at 37°C with 50 μM cephalothin and six concentrations of taniborbactam (0.125, 0.18, 0.26, 0.37, 0.52, and 0.75 μM). For CTX-M-15, reactions were initiated by addition of 3 pmol of enzyme and were performed in quadruplicate at 37°C with 70 μM cephalothin and six concentrations of taniborbactam (0.75, 0.915, 1.12, 1.36, 1.66, and 2.03 μM). Similar concentration ranges were tested for both avibactam and vaborbactam. A reversible two-step inhibition model was fit to the data.

Pseudo-first-order rate constants of enzyme inactivation (*k*_obs_) were determined in the presence of various inhibitor concentrations by fitting equation 2 to the time courses.

(2) $$P=V_{s}t+(V_{0}-V_{s})\frac{\left(1-e^{-\mathrm{kt}}\right)}{k}$$

where *P* is product, *V_s_* is rate at steady state, *t* is time, *V*_0_ is initial velocity, and *k* is rate of inactivation. A plot of *k*_obs_ versus the inhibitor concentrations generated a linear plot with a slope of (*k*_2_/*K_i_*) and a *y* intercept of *k*_−2_ (equation 3). The reported values were corrected for the substrate concentration and the *K_m_* for each substrate-enzyme combination, as defined by the 1 + ([*S*]/*K_m_*) term in equation 4, where *S* is the substrate concentration, used to obtain the second-order rate constant *k*_2_/*K_i_* and the off rate (*k*_−2_). The error values reported are standard errors from the fit.

(3) kobs=k−2+(k2/Ki)[I][1+([S]/Km)]

A plot of the fractional steady-state velocities showed a linear relationship with the inhibitor concentrations tested, from which an equilibrium dissociation constant ($K_{i}^{*}$; equation 4) was derived (equation 3).

(4) $$K_{i}^{*}=\frac{K_{i}k_{-2}}{k_{2}+k_{-2}}$$

Imipenem and cefotaxime were used as substrates for determining the $K_{i}^{*}$ of taniborbactam with OXA-48 and SHV-5, respectively. Taniborbactam was tested in triplicate at concentrations ranging from 0.050 to 5 μM with OXA-48 (2.5 nM) and from 0.4 to 100 nM with SHV-5 (5 nM) in 50 mM sodium phosphate (pH 7.0). Similar concentration ranges were tested with avibactam and vaborbactam.

Off rates (*k*_−2_) were assessed by a jump dilution method in triplicate reactions monitored continuously for the recovery of nitrocefinase enzymatic activity (Δε, −20,500 M^−1^ cm^−1^ at 486 nm). CTX-M-15 (2 μM), KPC-2 (4 μM), and P99 AmpC (1 μM) were inactivated by taniborbactam at 10, 20, and 5 μM, respectively. Similar concentration ranges were tested with avibactam and vaborbactam. A 4,000-fold jump dilution was performed for the β-lactamase–taniborbactam reaction mixtures. Enzyme activity was monitored continuously by determination of the absorbance at 486 nm after the final 10-fold dilution, consisting of the addition of 20 μl of an inactivation reaction mixture to 180 μl of 1× phosphate-buffered saline, pH 7.4, 0.1 mg/ml BSA at 37°C and nitrocefin at a 150 μM final concentration. The percentage of enzyme activity at each assay point was determined by comparison to the activity of the enzyme in the absence of inhibitor, and the recovery of enzymatic activity was fit to a single exponential with the associated standard deviation.

### Inhibition of VIM-2 and NDM-1 MBLs.

The inhibition modality of taniborbactam with both the VIM-2 and NDM-1 metallo-β-lactamases was assessed by monitoring the impact of various concentrations of taniborbactam on the Michaelis-Menten kinetic parameters *K_m_* and *V*_max_. The time dependence of the inhibitory activity was examined by preincubation of the enzyme with the inhibitor for up to 30 min prior to the initiation of the reactions, and the recovery of enzymatic activity was assessed by the jump dilution method, as previously described (33). For VIM-2, triplicate reactions (150 μl) were initiated by addition of the substrate nitrocefin, which was present at concentrations ranging from 11.5 to 200 μM. The reaction mixture contained 141 ng (4.5 pmol) of VIM-2 tested against five concentrations of taniborbactam (5, 10, 20, 40, and 80 nM), and substrate hydrolysis was monitored at 486 nm using a BioTek PowerWave XS2 UV/visible spectrophotometric plate reader. In contrast, for NDM-1, triplicate reactions (300 μl) were initiated by addition of the substrate cefotaxime, which was present at concentrations ranging from 13.1 to 150 μM. The reaction mixture contained 622.4 ng (24 pmol) of NDM-1 tested against seven concentrations of taniborbactam (6, 12, 24, 48, 72, 96, and 120 nM), and substrate hydrolysis was monitored at 260 nm. Avibactam and vaborbactam showed no significant inhibition up to 50 μM. Nitrocefin was used as the substrate for assays with IMP-1. No preincubation of the enzyme and inhibitor was required for either VIM-2 or NDM-1. The inhibition modality was ascertained by global fitting of the Michaelis-Menten equation for competitive inhibition (equation 5) to all the data, from which the *K_i_* values obtained with VIM-2 and NDM-1 were calculated.

(5) $$V_{0}=\frac{V_{\max\;}[S]}{\left[S\right]+K_{m}\left[1+\left(\left[I\right]/K_{i}\right)\right]}$$

where [*I*] is the inhibitor concentration.

### Antibacterial activity.

The *in vitro* antibacterial activity of the β-lactams alone or in combination with the BLIs was determined in cation-adjusted Mueller-Hinton broth (CAMHB) microdilution assays according to CLSI recommendations (51). The potentiation of antibacterial activity by taniborbactam, avibactam, or tazobactam was assayed by fixing the concentration of the BLI at 4 μg/ml for cefepime, ceftazidime, and ceftolozane, while tazobactam was added at a fixed concentration of 8 μg/ml to cefepime, as described by Wockhardt Pharmaceuticals. The inocula for the broth microdilution assays were prepared by the broth culture method (51). The β-lactams were 2-fold serially diluted in CAMHB, with the final concentrations generally ranging from 0.06 to 128 μg/ml when the agents were tested alone and from 0.016 to 32 μg/ml when they were tested in combination with their respective BLI. The MICs reported are modal values from 5 independent replicates performed on separate days either in engineered E. coli DH5α producing individual β-lactamases or in clinical isolates of *Enterobacterales* and P. aeruginosa.

### Justification for use of a fixed concentration of 4 μg/ml of taniborbactam for *in vitro* testing.

The *in vitro* activity of cefepime-taniborbactam was measured as the activity of cefepime in the presence of taniborbactam at a fixed concentration of 4 μg/ml. The rationale for choosing 4 μg/ml of taniborbactam for *in vitro* testing was based on *in vitro* assessments by broth microdilution and time-kill assays, the correlation of the results obtained with nonclinical models of infection, and pharmacokinetic-pharmacodynamic modeling.

Taniborbactam alone lacks antibacterial activity. At a fixed concentration of 4 μg/ml taniborbactam, the MIC_90_ of cefepime was reduced from >128 to 2 μg/ml for cefepime-nonsusceptible *Enterobacterales* isolates, with 97.6% of the isolates being inhibited by 8 μg/ml of cefepime (52). This value corresponds to the susceptible, dose-dependent (SDD) breakpoint for cefepime (37). The MIC distribution of cefepime-taniborbactam with taniborbactam fixed at 4 μg/ml against cefepime-nonsusceptible *Enterobacterales* from surveillance similarly resembles the MIC distribution of cefepime against cefepime-susceptible isolates from the same study (52). The overlapping MIC distributions for cefepime against cefepime-susceptible strains and cefepime-taniborbactam against cefepime-nonsusceptible strains (with taniborbactam fixed at 4 μg/ml) reflect the complete or nearly complete rescue of the activity of cefepime by taniborbactam against target organisms. Importantly, humanized dosing of cefepime and taniborbactam in the neutropenic murine thigh infection model reduced the bacterial burden of all isolates of *Enterobacterales* and P. aeruginosa with cefepime-taniborbactam MIC values of 8 μg/ml or below by at least 1 log_10_ (53). These results are consistent with the MIC values for cefepime derived in the presence of 4 μg/ml of taniborbactam.

### Isolation of avibactam and ceftolozane from clinical preparations.

Avibactam was isolated from commercially available ceftazidime-avibactam (Avycaz). A single bottle of Avycaz (containing 2 g of ceftazidime and 0.5 g of avibactam) was dissolved in approximately 6 to 8 ml of water and purified using a Biotage 120-g C_18_ reverse-phase column. A gradient utilizing a 99:1 mixture of H_2_O (with 0.1% trifluoroacetic acid [TFA])-acetonitrile (ACN) (with 0.1% TFA) for four column volumes (CV), followed by a ramp to 1:100 H_2_O-ACN over 1 CV, was employed. All fractions containing avibactam were combined, frozen, and lyophilized to provide pure avibactam. Electrospray ionization mass spectrometry (ESI-MS) confirmed an *m/z* of 266.1 (M + H)^+^.

Ceftolozane was isolated from commercially available ceftolozane-tazobactam (Zerbaxa). A single bottle of Zerbaxa (containing 1 g of ceftolozane and 0.5 g tazobactam) was dissolved in water (∼6 to 8 ml) and purified using a Biotage Snap Ultra 120-g C_18_ reverse-phase column. A gradient beginning with 5 CVs of 95:5 H_2_O (with 0.1% TFA)-ACN (with 0.1% TFA) was followed by a ramp to 80:20 H_2_O-ACN over 6 CVs. The collected fractions were combined, frozen, and lyophilized. The isolated solid was triturated with 4.0 N HCl in diethyl ether and concentrated three times to provide ceftolozane as the hydrochloride salt. ESI-MS confirmed an *m/z* of 667.1 (M + H)^+^.

### *In vitro* antibacterial kill assay.

Bactericidal activity was assessed by a time-kill assay according to standard CLSI methods (54). The cefepime concentrations to be assayed in combination with taniborbactam were selected based on antibacterial activity (MIC), while ceftazidime-avibactam was tested at 32 μg/ml (which is 4 times the CLSI susceptibility breakpoint of 8 μg/ml) (37). Time-kill assays were performed in 14-ml glass tubes with a bacterial inoculum in CAMHB of 5 × 10^5^ CFU/ml. The tubes were incubated at 37°C with shaking at 200 rpm, and aliquots were drawn at six time points (0, 2, 4, 6, 8, and 24 h) to prepare 0.5-log_10_ dilutions in CAMHB in 96-well plates with activated charcoal. From these dilution plates, 10 μl was spotted onto OmniTrays using a replicator. Both the OmniTrays and the dilution plates were incubated overnight at 37°C and used to ascertain the viable bacterial counts. The smallest quantifiable amount obtained by this method was 2 log_10_ CFU.

### Spontaneous frequency of resistance.

Five colonies were picked from agar plates with an inoculating loop and transferred aseptically to a glass Erlenmeyer flask containing 20 ml of CAMHB. The culture was grown for ≥5 h at 37°C with shaking at 200 rpm. CAMHB agar (15 g/liter; Difco agar; Becton, Dickinson) was prepared according to the manufacturer’s directions. Cefepime was added at 4× the MIC along with taniborbactam at a fixed concentration of 4 μg/ml. The agar was dispensed into 100-mm petri dishes (∼20 ml per dish), with 100 plates being used per test organism. Agar plates without drug were used to obtain colony counts. The optical density of the growing cultures used for inoculation was assessed spectrophotometrically at 600 nm. The inocula were adjusted to a final suspension of 1 × 10^8^ CFU/ml to 1 × 10^9^ CFU/ml. The inoculum (100 μl) was spread onto each plate with an inoculating loop. The plates were incubated at 37°C, and colony counts were assessed at 24 and 48 h.

### Data availability.

The GenBank accession numbers for the individual expression plasmids are MN307371, MN401186, MN401152 to MN401166, and MN401168 to MN401185.

## Supplementary Material

Supplemental file 1
