# Supplementary material for: VNRX-5133 (Taniborbactam), a Broad-Spectrum Inhibitor of Serine- and Metallo-β-Lactamases, Restores Activity of Cefepime in Enterobacterales and Pseudomonas aeruginosa
Source: Antimicrob Agents Chemother. 2020 Feb 21;64(3):e01963-19. doi: 10.1128/AAC.01963-19 (PMC7038240; doi:10.1128/AAC.01963-19)
Supplement: Supplemental file 1 [file AAC.01963-19-s0001.pdf]

Supplemental Material

VNRX-5133 (Taniborbactam), a broad spectrum inhibitor of serine- and metallo-β-lactamases, restores activity of cefepime in *Enterobacterales* and *P. aeruginosa.*

Jodie C. Hamrick, Jean-Denis Docquier[a], Tsuyoshi Uehara, Cullen L. Myers, David A. Six, Cassandra L. Chatwin, Kaitlyn J. John, Salvador F. Vernacchio, Susan M. Cusick, Robert E.L. Trout, Cecilia Pozzi[b], Filomena De Luca[a], Manuela Benvenuti[b], Stefano Mangani[b], Bin Liu, Randy W. Jackson, Greg Moeck, Luigi Xerri, Christopher J. Burns, Daniel C. Pevear and Denis M. Daigle[#]

VenatoRx Pharmaceuticals Incorporated, Malvern Pennsylvania, USA and [a]Department of Medical Biotechnology, [b]Department of Biotechnology, Chemistry and Pharmacy, University of Siena, Siena, Italy.

TABLE S1. Expanded Microbiological profiling of cefepime-taniborbactam and other cephalosporin-BLI combinations in BL resistant gram-negatives.

			**Minimal Inhibitory Concentration (µg/mL)**						
Species	**Strain ID**	**Molecular Summary**	**FEP-TAN**	**FEP-TZB**	**FEP**	**CZA**	**CAZ**	**C/T**	**TOL**
**Enterobacteriaceae**									
**Mixed Class A and C (n = 22)**									
K. aerogenes	73546	AmpC	0.03	0.03	0.5	0.12	32	1	4
K. aerogenes	110099	AmpC/SHV-12	0.25	0.5	4	1	128	16	64
K. aerogenes	111707	AmpC/TEM-1	≤ 0.016	0.03	4	0.5	32	0.5	4
E. cloacae	20	p99 AmpC/CTX-M-27/ SHV-12/ACT-2/ TEM-135	0.06	0.06	4	1	≥ 256	1	32
E. cloacae	116723	p99 AmpC/TEM-1/ CTX-M-3	0.5	0.5	4	1	128	4	32
E. cloacae	138212	p99 AmpC/SHV-71/ TEM-1	0.06	0.06	2	0.5	128	16	32

Species	Strain ID	Molecular Summary	Minimal Inhibitory Concentration (µg/mL)						
			FEP-TAN	FEP-TZB	FEP	CZA	CAZ	C/T	TOL
E. coli	469	AmpC/SHV-12/TEM-1	0.03	0.03	1	0.25	64	0.25	8
E. coli	EC1552 2A	AmpC/CMY-2/TEM-1, CTX-M-15	0.5	1	4	0.25	64	8	16
E. coli	SI-PO26	AmpC/CMY-2/TEM-1	0.03	0.06	0.25	0.25	64	4	8
K. oxytoca	134943	CMY-2/TEM-1	0.06	0.5	4	1	128	8	8
K. pneumoniae	178279	SHV-12/TEM-1/AmpC	0.03	0.06	8	1	128	32	64
K. pneumoniae	196477	AmpC	0.06	2	16	4	≥ 256	≥ 64	≥ 256
K. pneumoniae	212027	AmpC/FOX-5/TEM-1	0.06	0.25	2	4	128	8	8
K. pneumoniae	SI-F100	SHV-5/TEM-1/CMY-2	0.03	0.03	1	0.25	32	0.5	16
S. marcescens	134434	AmpC/SHV-12/TEM-1	0.06	0.12	4	0.5	64	2	64
S. marcescens	192476	AmpC/TEM-1/SHV-5	0.12	1	4	0.5	128	≥ 64	64
S. marcescens	192478	AmpC/SHV-12/TEM-1	0.06	0.06	4	0.5	128	32	64

Species	Strain ID	Molecular Summary	Minimal Inhibitory Concentration (µg/mL)						
			FEP-TAN	FEP-TZB	FEP	CZA	CAZ	C/T	TOL
S. marcescens	218073	AmpC/SHV-7/TEM-1	0.06	0.5	2	0.5	64	32	64
S. typhi	26A	AmpC/TEM-1	0.06	0.06	4	0.5	64	0.5	16
S. paratyphi	527	AmpC/TEM-1	0.06	0.06	64	0.5	8	2	32
C. freundii	134390	AmpC/TEM-1	≤ 0.016	0.12	0.5	0.12	16	0.25	8
C. freundii	241181	AmpC/TEM-163	≤ 0.016	0.03	8	0.25	8	0.5	16
		**% Susceptible isolates[1]**	**100**	**100**	**90.9**	**100**	**9.1**	**40.9**	**0**
**OXA-48/48-like (n = 20)**									
E. cloacae	976004	OXA-48	0.5	16	128	1	128	≥ 64	≥ 256
E. coli	664507	OXA-48	0.25	2	16	0.25	64	16	64
E. coli	664516	OXA-48	0.5	4	64	0.5	64	32	64
E. coli	664520	OXA-48	0.12	1	32	0.25	64	16	64

Species	Strain ID	Molecular Summary	Minimal Inhibitory Concentration (µg/mL)						
			FEP-TAN	FEP-TZB	FEP	CZA	CAZ	C/T	TOL
E. coli	664522	OXA-48	0.5	2	64	0.5	64	32	128
E. coli	664523	OXA-48	0.12	1	128	0.25	64	16	64
E. coli	VER	OXA-48	0.06	0.25	0.5	0.12	0.5	1	2
K. pneumoniae	6299	OXA-163	0.25	≥ 64	64	2	128	≥ 64	≥ 256
K. pneumoniae	515746	OXA-48	0.25	1	32	0.25	64	16	128
K. pneumoniae	664438	OXA-48	0.25	4	32	0.5	128	≥ 64	128
K. pneumoniae	664439	OXA-48	0.25	4	32	0.5	128	32	128
K. pneumoniae	722870	OXA-48	0.25	2	8	0.5	64	32	64
K. pneumoniae	752334	OXA-48	0.06	1	128	0.5	128	≥ 64	≥ 256
K. pneumoniae	769972	OXA-48	0.12	1	16	0.5	64	32	128
K. pneumoniae	787422	OXA-48	0.25	4	64	1	8	32	64

Species	Strain ID	Molecular Summary	Minimal Inhibitory Concentration (µg/mL)						
			FEP-TAN	FEP-TZB	FEP	CZA	CAZ	C/T	TOL
K. pneumoniae	892694	SHV-12/TEM-1/CTX-M-15/OXA-48/ACT	0.5	4	32	1	≥ 256	≥ 64	≥ 256
K. pneumoniae	DOV	OXA-48	0.12	0.25	32	0.25	64	8	128
K. pneumoniae	KIRK	OXA-162	0.25	2	16	0.5	128	32	128
K. pneumoniae	SI-C05	OXA-48	0.12	1	16	0.5	64	32	≥ 256
M. morgannii	769909	OXA-48	0.06	0.06	0.06	0.12	2	1	8
		**% Susceptible isolates[1]**	**100**	**90**	**15**	**100**	**15**	**10**	**5**
**ESBL (n = 20)**									
E. cloacae	874381	CTX-M-15	1	2	≥ 256	1	≥ 256	≥ 64	≥ 256
E. cloacae	875134	TEM-1/CTX-M-3/DHA-1	0.5	1	128	0.5	64	16	64
E. cloacae	891849	TEM-1	0.12	1	4	1	128	16	32

Species	Strain ID	Molecular Summary	Minimal Inhibitory Concentration (μg/mL)						
			FEP-TAN	FEP-TZB	FEP	CZA	CAZ	C/T	TOL
E. cloacae	937223	SHV-12/CTX-M-9/ACT-7	1	1	16	1	≥ 256	4	32
E. cloacae	938772	TEM-OSBL/CTX-M-15/ACT-30	1	1	128	2	128	16	128
E. coli	1924	TEM-12	0.06	0.03	16	0.5	32	0.5	16
E. coli	873435	CTX-M-15	0.06	0.06	128	0.5	128	2	128
E. coli	875627	CTX-M-27	0.03	0.03	8	0.25	8	0.5	16
E. coli	878358	TEM-1/CTX-M	0.12	0.12	≥ 256	1	32	16	64
E. coli	890500	TEM-1/CTX-M-14	0.06	0.03	64	0.25	4	0.5	8
E. coli	ESBL 5	CTX-M-15/TEM-1	0.5	0.12	≥ 256	0.5	128	1	128
E. coli	SI-LP377	CTX-M-2	0.03	0.03	64	0.12	8	1	32
E. coli	SI-M004	SHV-2/GES-12	0.03	0.12	16	0.5	≥ 256	8	64

			Minimal Inhibitory Concentration (µg/mL)						
Species	**Strain ID**	**Molecular Summary**	**FEP-TAN**	**FEP-TZB**	**FEP**	**CZA**	**CAZ**	**C/T**	**TOL**
E. coli	SI-V502	TEM-29/CTX-M-15	0.12	0.25	8	0.5	32	1	8
K. pneumoniae	873460	SHV-30/CTX-M-15	0.12	0.12	32	0.5	64	2	128
K. pneumoniae	ESBL8 #1	TEM-1/SHV-12	≤0.016	0.25	4	0.12	≥ 256	8	32
K. pneumoniae	ESBL 10	SHV-12/TEM-1	0.12	0.06	8	0.5	128	4	32
K. pneumoniae	KP 3	SHV-1/TEM-1	0.12	4	16	2	≥ 256	≥64	≥ 256
K. pneumoniae	KPN 508	SHV-7/TEM-1	0.06	0.06	8	1	≥ 256	16	32
K. pneumoniae	SI-KP NO30	TEM-26b/SHV-60/ SHV-26	0.06	0.03	16	0.5	≥ 256	0.5	64
		**% Susceptible isolates[1]**	**100**	**100**	**30**	**100**	**15**	**45**	**0**
**KPC (n = 20)**									
E. cloacae	847426	KPC	1	≥ 64	≥ 256	2	≥ 256	≥ 64	≥ 256
E. cloacae	CDC-0053	KPC-3/TEM-1	0.25	32	128	2	≥ 256	≥ 64	128

Species	Strain ID	Molecular Summary	Minimal Inhibitory Concentration (µg/mL)						
			FEP-TAN	FEP-TZB	FEP	CZA	CAZ	C/T	TOL
E. cloacae	CDC-0163	KPC/CTX-M-1/ ACT/MIR	1	8	64	1	64	32	128
E. coli	849121	KPC	0.06	8	64	0.5	64	32	32
E. coli	CDC-0001	KPC	1	16	128	1	≥ 256	32	64
K. oxytoca	847537	KPC	0.25	32	32	16	≥ 256	≥ 64	≥ 256
K. pneumoniae	137052	SHV-11/KPC-3/ TEM-1	0.5	16	16	2	128	≥ 64	64
K. pneumoniae	845661	KPC	1	≥ 64	≥ 256	4	≥ 256	≥ 64	≥ 256
K. pneumoniae	847204	KPC	0.12	16	32	2	64	≥ 64	64
K. pneumoniae	848562	KPC	1	≥ 64	≥ 256	8	≥ 256	≥ 64	≥ 256
K. pneumoniae	848600	KPC	1	≥ 64	≥ 256	4	≥ 256	≥ 64	≥ 256
K. pneumoniae	848828	KPC	1	≥ 64	≥ 256	2	≥ 256	≥ 64	128

			Minimal Inhibitory Concentration (µg/mL)						
Species	**Strain ID**	**Molecular Summary**	**FEP-TAN**	**FEP-TZB**	**FEP**	**CZA**	**CAZ**	**C/T**	**TOL**
K. pneumoniae	849170	KPC	0.5	≥ 64	≥ 256	2	≥ 256	≥ 64	128
K. pneumoniae	864085	KPC	0.12	8	32	8	≥ 256	≥ 64	≥ 256
K. pneumoniae	871489	KPC	1	≥ 64	≥ 256	8	≥ 256	≥ 64	≥ 256
K. pneumoniae	882752	SHV-11/TEM-1/ KPC-3	0.5	≥ 64	128	1	≥ 256	≥ 64	128
K. pneumoniae	884679	KPC	0.06	8	32	1	≥ 256	≥ 64	64
K. pneumoniae	CDC-0003	KPC	0.12	16	32	4	≥ 256	≥ 64	128
K. pneumoniae	CDC-0117	KPC-3	0.5	≥ 64	64	2	≥ 256	≥ 64	128
K. pneumoniae	CDC-0125	KPC-3	0.12	32	64	4	≥ 256	≥ 64	128
		**% Susceptible isolates[1]**	**100**	**20**	**0**	**95**	**0**	**0**	**0**
**MBL (n = 20)**									
E. cloacae	920937	VIM-1	4	≥ 64	128	≥ 64	≥ 256	≥ 64	≥ 256

Species	Strain ID	Molecular Summary	Minimal Inhibitory Concentration (μg/mL)						
			FEP-TAN	FEP-TZB	FEP	CZA	CAZ	C/T	TOL
E. cloacae	1249072	TEM-OSBL(b)/VIM-1	1	≥ 64	32	≥ 64	≥ 256	≥ 64	≥ 256
E. cloacae	1365477	TEM-OSBL(b)/VIM-1	0.5	32	32	≥ 64	≥ 256	≥ 64	≥ 256
E. coli	1275635	VIM-23	0.5	4	64	≥ 64	128	≥ 64	≥ 256
E. coli	1459453	NDM-7	1	≥ 64	≥ 256	≥ 64	≥ 256	≥ 64	≥ 256
E. coli	CDC-0069	NDM	0.25	≥ 64	32	≥ 64	≥ 256	≥ 64	≥ 256
E. coli	CDC-0149	NDM	2	≥ 64	≥ 256	≥ 64	≥ 256	≥ 64	≥ 256
K. pneumoniae	856819	SHV-11/VIM-1	0.12	32	32	≥ 64	≥ 256	≥ 64	≥ 256
K. pneumoniae	874328	SHV-11/CTX-M-15/ NDM-1	2	≥ 64	128	≥ 64	≥ 256	≥ 64	≥ 256
K. pneumoniae	882956	SHV-31/DHA-1/VIM-1	0.12	32	16	≥ 64	≥ 256	≥ 64	≥ 256
K. pneumoniae	890066	SHV-33/TEM-33/ CTX-M-15/IMP-1	8	16	64	≥ 64	≥ 256	≥ 64	≥ 256

Species	Strain ID	Molecular Summary	Minimal Inhibitory Concentration (µg/mL)						
			FEP-TAN	FEP-TZB	FEP	CZA	CAZ	C/T	TOL
K. pneumoniae	919877	SHV-OSBL/VIM-1	0.25	≥ 64	128	≥ 64	≥ 256	≥ 64	≥ 256
K. pneumoniae	1104864	SHV-OSBL(b)/VIM-1	2	≥ 64	≥ 256	≥ 64	≥ 256	≥ 64	≥ 256
K. pneumoniae	1104866	SHV-OSBL(b)/VIM-1	1	≥ 64	≥ 256	≥ 64	≥ 256	≥ 64	≥ 256
K. pneumoniae	1280442	SHV-OSBL(b)/VIM-1	0.12	≥ 64	16	≥ 64	≥ 256	≥ 64	≥ 256
K. pneumoniae	CDC-0049	NDM	4	≥ 64	≥ 256	≥ 64	≥ 256	≥ 64	≥ 256
K. pneumoniae	CDC-0139	NDM-1	1	≥ 64	≥ 256	≥ 64	≥ 256	≥ 64	≥ 256
K. pneumoniae	CDC-0145	NDM	0.5	≥ 64	≥ 256	≥ 64	≥ 256	≥ 64	≥ 256
K. pneumoniae	CDC-0148	NDM	2	≥ 64	128	≥ 64	≥ 256	≥ 64	≥ 256
K. pneumoniae	CDC-0152	NDM	0.5	≥ 64	≥ 256	≥ 64	≥ 256	≥ 64	≥ 256
		**% Susceptible isolates[1]**	**100**	**5**	**0**	**0**	**0**	**0**	**0**

P. aeruginosa

Wild-type, basal level Class C and OprD downregulation with RND efflux upregulation (n=14)

Species	Strain ID	Molecular Summary	Minimal Inhibitory Concentration (μg/mL)						
			FEP-TAN	FEP-TZB	FEP	CZA	CAZ	C/T	TOL
P. aeruginosa	1129	PDC	4	4	4	4	4	1	1
P. aeruginosa	3615	PDC/TEM-1	1	8	16	4	32	4	4
P. aeruginosa	8273	PDC/TEM-1	1	8	8	4	16	2	1
P. aeruginosa	164951	W.T.	1	1	1	2	2	0.5	1
P. aeruginosa	377860	W.T.	4	4	4	2	4	1	1
P. aeruginosa	384743	PDC/TEM-1	4	8	4	4	16	2	16
P. aeruginosa	ATCC 27853	W.T.	1	1	1	2	2	0.5	1
P. aeruginosa	PA2	W.T.	4	4	8	2	4	1	2
P. aeruginosa	Paeβ-04	100-fold ↑ PDC-2/30% OprD/5.6x ↑ABM	4	16	16	8	32	2	2

Species	Strain ID	Molecular Summary	Minimal Inhibitory Concentration (µg/mL)						
			FEP-TAN	FEP-TZB	FEP	CZA	CAZ	C/T	TOL
P. aeruginosa	Paeβ-10	67-fold ↑ PDC-2/0.1% OprD/5.7x↑ABM	8	16	16	4	32	2	2
P. aeruginosa	Paeβ-15	310-fold ↑ PDC-2/10% OprD/4.4x↑XYM	4	32	128	4	64	4	4
P. aeruginosa	Paeβ-17	62-fold ↑PDC-3/30% OprD/10.2x and 9.9x ↑XYM and CDJ	2	8	16	8	64	2	2
P. aeruginosa	Paeβ-18	600-fold ↑ PDC-3/ <0.01% OprD/3.9x and 5.6x ↑ABM and XYM	32	≥ 64	64	≥ 64	≥ 256	16	16
P. aeruginosa	Paeβ-21	200-fold ↑ PDC-4/10% OprD/13.5x ↑XYM	4	8	16	4	64	2	2
		**% Susceptible isolates[1]**	**92.9**	**71.4**	**50**	**92.9**	**35.7**	**92.9**	**85.7**
**PDC variants (n = 10)**									
P. aeruginosa	10.257	PDC-50	2	16	32	8	≥ 256	16	16

Species	Strain ID	Molecular Summary	Minimal Inhibitory Concentration (µg/mL)						
			FEP-TAN	FEP-TZB	FEP	CZA	CAZ	C/T	TOL
P. aeruginosa	13.1717	PDC-74	8	≥ 64	64	≥ 64	≥ 256	≥ 64	64
P. aeruginosa	13.1716	PDC-74	16	32	128	≥ 64	≥ 256	16	128
P. aeruginosa	13.1760	PDC-77	8	16	16	32	≥ 256	32	32
P. aeruginosa	11.516	PDC-79	8	16	16	≥ 64	128	≥ 64	64
P. aeruginosa	13.1737	PDC-80	4	16	16	16	128	≥ 64	128
P. aeruginosa	13.1755	PDC-86	4	16	16	≥ 64	≥ 256	≥ 64	128
P. aeruginosa	11.698	PDC-87	16	≥ 64	128	≥ 64	≥ 256	32	32
P. aeruginosa	13.1775	PDC-87	4	≥ 64	64	≥ 64	≥ 256	16	16
P. aeruginosa	11.813	PDC-91	8	≥ 64	≥ 256	≥ 64	≥ 256	8	8
		**% Susceptible isolates[1]**	**80**	**0**	**0**	**10**	**0**	**0**	**0**

Species	Strain ID	Molecular Summary	Minimal Inhibitory Concentration (µg/mL)						
			FEP-TAN	FEP-TZB	FEP	CZA	CAZ	C/T	TOL
**GES/KPC/VIM carbapenemase producing (n = 17)**									
P. aeruginosa	1013996	KPC-2	2	≥ 64	≥ 256	4	64	16	16
P. aeruginosa	1071703	GES-6	4	8	16	4	64	≥ 64	32
P. aeruginosa	1113755	GES-5	4	32	32	16	≥ 256	≥ 64	≥ 256
P. aeruginosa	1138223	GES-19	4	≥ 64	≥ 256	≥ 64	≥ 256	≥ 64	≥ 256
P. aeruginosa	1138337	GES-19	4	≥ 64	≥ 256	32	≥ 256	≥ 64	≥ 256
P. aeruginosa	1258509	GES-6	8	16	32	4	64	≥ 64	64
P. aeruginosa	1275655	GES-20	1	≥ 64	≥ 256	≥ 64	≥ 256	≥ 64	≥ 256
P. aeruginosa	1300089	GES-19	8	≥ 64	≥ 256	≥ 64	≥ 256	≥ 64	≥ 256
P. aeruginosa	1313666	KPC-2	2	≥ 64	≥ 256	4	64	32	32
P. aeruginosa	CDC-0090	KPC	4	≥ 64	128	4	≥ 256	32	32
P. aeruginosa	PAA1-9G1	GES-5	8	≥ 64	≥ 256	≥ 64	≥ 256	≥ 64	≥ 256

			Minimal Inhibitory Concentration (µg/mL)						
Species	**Strain ID**	**Molecular Summary**	**FEP-TAN**	**FEP-TZB**	**FEP**	**CZA**	**CAZ**	**C/T**	**TOL**
P. aeruginosa	PAA1-9G2	GES-5	8	≥ 64	≥ 256	≥ 64	≥ 256	≥ 64	≥ 256
P. aeruginosa	247.3D	VIM-2	8	32	32	≥ 64	≥ 256	≥ 64	≥ 256
P. aeruginosa	5671	VIM-1	2	16	16	≥ 64	128	≥ 64	64
P. aeruginosa	PS-12	VIM-2	4	32	32	≥ 64	64	≥ 64	≥ 256
P. aeruginosa	PS-32	VIM-2	4	16	8	32	16	≥ 64	128
P. aeruginosa	Ps296	VIM-2	1	4	4	32	64	≥ 64	≥ 256
		**% Susceptible isolates[1]**	**100**	**11.8**	**5.9**	**29.4**	**0**	**0**	**0**

Abbreviations: µg/mL = microgram, AVI = avibactam, CAZ = ceftazidime, TOL = ceftolozane FEP = cefepime, mL = milliliter, TZB = tazobactam, TAN = taniborbactam. All β-lactamase inhibitors were tested at a fixed concentration of 4 µg/ml with the exception of tazobactam in combination with cefepime where BLI was fixed at 8 µg/mL. Modal MIC values are reported from a minimum of 4 replicates.[1]Percent (%) susceptibility was defined according to CLSI SDD interpretive criteria for cefepime (MIC ≤ 8 µg/mL) and this value was used for cefepime-tazobactam and cefepime alone; percent susceptibility for ceftazidime was defined as CLSI interpretive criteria for ceftazidime-avibactam (MIC ≤ 8 µg/mL); ceftolozane alone and ceftolozane-tazobactam used the CLSI interpretive criteria for ceftolozane-tazobactam of MIC ≤ 2 µg/mL in Enterobacterales and MIC ≤ 4 µg/mL

in *P. aeruginosa*. Since breakpoints are not yet available for cefepime-taniborbactam, %S refers to proportion of tested isolates inhibited at or below 8 µg/mL which corresponds to the cefepime SDD breakpoint (CLSI M100) and %R refers to the proportion of tested isolates with cefepime-taniborbactam MICs ≥16 µg/mL.

TABLE S2. Plasmids for expression of β-lactamases in *E. coli*

Plasmid name	Encoded enzyme	GenBank accession number
pTU501	-	MN307371
pTU502	NDM-1	MN401186
pTU504	NDM-5	MN401152
pTU505	NDM-7	MN401153
pTU512	VIM-1	MN401154
pTU513	VIM-2	MN401155
pTU514	VIM-4	MN401156
pTU525	SPM-1	MN401157
pTU526	GIM-1	MN401158
pTU535	IMP-1	MN401159
pTU556	TEM-10	MN401160
pTU558	TEM-24	MN401161
pTU559	TEM-72	MN401162
pTU560	CTX-M-2	MN401163
pTU561	CTX-M-15	MN401164

Plasmid name	Encoded enzyme	GenBank accession number
pTU562	GES-5	MN401165
pTU563	SHV-5	MN401166
pTU565	VEB-9	MN401168
pTU566	KPC-2	MN401169
pTU567	KPC-3	MN401170
pTU569	KPC-3(D179Y)	MN401171
pTU570	KPC-3(V240G)	MN401172
pTU571	KPC-3(T243A)	MN401173
pTU572	KPC-3(A177E/D179Y)	MN401174
pTU573	KPC-3(D179Y/T243M)	MN401175
pTU576	CMY-2	MN401176
pTU578	ACT-C189	MN401177
pTU579	ACT-17	MN401178
pTU581	OXA-48	MN401179
pTU582	OXA-162	MN401180
pTU583	OXA-163	MN401181

Plasmid name	Encoded enzyme	GenBank accession number
pTU584	OXA-181	MN401182
pTU586	OXA-232	MN401183
pTU595	PER-1	MN401184
pTU596	PER-2	MN401185
